# Low-power artificial neuron networks with enhanced synaptic functionality using dual transistor and dual memristor

**DOI:** 10.1371/journal.pone.0318009

**Published:** 2025-01-27

**Authors:** Keerthi Nalliboyina, Sakthivel Ramachandran

**Affiliations:** School of Electronic Science Engineering, Vellore Institute of Technology, Vellore, India; Lanzhou University of Technology, CHINA

## Abstract

Artificial neurons with bio-inspired firing patterns have the potential to significantly improve the performance of neural network computing. The most significant component of an artificial neuron circuit is a large amount of energy consumption. Recent literature has proposed memristors as a promising option for synaptic implementation. In contrast, implementing memristive circuitry through neuron hardware presents significant challenges and is a relevant research topic. This paper describes an efficient circuit-level mixed CMOS memristor artificial neuron network with a memristor synapse model. From this perspective, the paper describes the design of artificial neurons in standard CMOS technology with low power utilization. The neuron circuit response is a modified version of the Morris-Lecar theoretical model. The suggested circuit employs memristor-based artificial neurons with Dual Transistor and Dual Memristor (DTDM) synapse circuit. The proposed neuron network produces a high spiking frequency and low power consumption. According to our research, a memristor-based Morris Lecar (ML) neuron with a DTDM synapse circuit consumes 12.55 pW of power, the spiking frequency is 22.72 kHz, and 2.13 fJ of energy per spike. The simulations were carried out using the Spectre tool with 45 nm CMOS technology.

## Introduction

In the developing field of neuromorphic computing, brain-inspired architectures like Artificial Neural Networks (ANNs) have been implemented using VLSI technologies [1]. On the other hand, real-time apps are carried out on mobile consumer platforms, and run on the edge computing applications. In wireless communications, such as 5G, fill the target application gap and can handle robust remote machine learning systems [2]. However, the development of more data and models means that there will always be a need for more targeted computing platforms. The intention is to deploy the mobile platform with an artificial intelligence learning model and conduct local prediction calculations.

**Fig 1:**
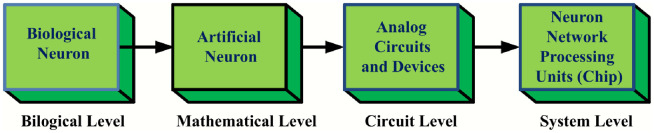
Design flow of bio-inspired artificial neurons.

Developing edge computing devices that are more efficient in speed and energy can be achieved through neuromorphic computing [3]. A design flow explaining the techniques used in neuromorphic engineering are in Fig 1. By abstracting the idea into a mathematical representation and applying it to the system level, one can take advantage of an effective natural process. A nerve cell is the basic building block of a neural system and is essential to its overall functionality.

The level of concentration of ions, such as calcium, potassium, and sodium, in the cell varies, and the membrane potential fluctuates. When an electromagnetic field is applied to the medium, polarization, and magnetization take place. This causes the electric field and charge distribution in the cells to be re-organized and controlled, which alters the membrane potential response. On the other hand, it suggests that the electrical activity of the brain and nervous system may be impacted by an external electromagnetic field. The perception that the brain is the most sophisticated neural system is supported by evidence illustrating the usefulness and potential of electromagnetic induction. Bifurcation was used to study the amplifying of memory in excitable systems [4].

From a physical perspective, a neuronal cell is a neural circuit that can be constructed using an inductor, capacitor, and any other necessary electric components. The inductor and capacitor can absorb external electromagnetic energy, and the interchange of field energy can vary the circuit’s outputs [5]. Consequently, the general circuit equation can be defined in Eqs 1 and 2.

$$\begin{array}{c} C_{m}\frac{dV}{d\tau }=F\left(V,i\right)+I_{ext} \end{array}$$ (1)

$$\begin{array}{c} L_{m}\frac{di}{d\tau }=G\left(V\right) \end{array}$$ (2)

where *i* is a recovery variable associated with transmembrane current and *V* denotes the membrane voltage. *I*_*ext*_ is an external force of the synaptic current. The current function, which depends on the cell membrane potential and recovery current, is denoted by the notation *F*(*V*, *i*). The voltage function related to the magnetic field is represented by *G*(*V*) [6].

The corresponding inductance of the neuron is *L*_*m*_, and membrane capacitance is *C*_*m*_, depends on the properties of the media. The magnetic field can be produced by any movement within the cell or by the continuous transmission of ions from dendrite to soma [7].

Many scientists have investigated the interactions between neurons in a network, in addition to focusing on the dynamic consequences of a single neuron. Chaos et al. examine the dynamics of a single neuron, subsequently analyzing the network derived from an enhanced FitzHugh-Nagumo model that incorporates memristive autapse [8]. Whenever membrane potential differences exist between two interconnected neurons in a neural system, inductive electromagnetic flows can be induced [9]. These effects are analogous for the bidirectional induced currents that emerge when a flux-controlled memristor links each two neurons [10].

The Moris Lecar (ML) model, which closely resembles the behavior of a biological neuron is a promising method to simplify the mechanisms of the neuron. Even the most straight forward tasks for humans, such as recognizing objects by their appearance under various conditions can be complex for computers [11]. As a feasible alternative to the current Von Neumann-based computing architecture and memory technology, a radical new device is known as the memristor. It has recently drawn the attention of researchers in academia and industry due to its promising prospects for upcoming neuromorphic computing applications. With its non-volatile property of retaining its present state as an instance of the memory resistance value [12].

Research has been conducted on the Spiking Neural Network (SNN), one of the neural networks, classified as a third-generation neural networks. The fundamental building blocks of signal processing in an SNN are its neuron circuits, which are artificial neuron models that resemble biological neurons and encode all signals using spikes according to either the rate or spiking time dependency method [13]. Consequently, the primary function of an SNN is spike generation, and many neurons with properties related to those of biological systems [14]. Suppressing dissipated power in a neuron’s circuits is the most crucial step towards reducing an SNN’s energy consumption. Consequently, some analog neuron circuit types have been proposed to implement artificial neuron circuits, including log domain neurons, Integrate-and-Fire (I&F) neurons, and conductance-based neurons, etc., [15]. The Wilson neuron model with a memristive autapse and the FitzHugh-Nagumo neuron model has been implemented using microcontroller [16, 17].

The ML model incorporates membrane potential fluctuations and ion channel dynamics to provide a simplified yet bio-physically meaningful representation of neuronal behavior [18]. The aim to create a hardware-efficient neuromorphic system that combines memristors with artificial neurons to emulate the ML model’s complex dynamics with minimal energy usage. This effort has many potential applications in edge computing, wearable technology, and Internet of Things (IoT)systems, where computational scalability and energy efficiency are critical factors [19].

This paper presents the design of an analog artificial neuron network (memristor based ML neuron model) using cadence virtuoso 45 nm CMOS technology. In introduction section, the fundamental ideas of biological neuron networks and artificial neuron network models are presented. The non-volatile memristor section described the VTEAM (Voltage ThrEshold Adaptive Memristor) model, which is used in this work. The different types of synapses i.e., Dual Transistor with One Memristor (DT1M) and Dual Transistor with Dual Memristor (DTDM) synapses are used in our proposed work and it is explained in the artificial synapses section. The next section explains, the proposed memristor-based artificial neuron with a DTDM synapse circuit. The comparison of our proposed design with existing neuron models is presented as different analysis of our proposed work has been discussed in the results and discussion section. Finally, the proposed work is summarized in the conclusion section.

## Brain-inspired artificial neuron

**Fig 2:**
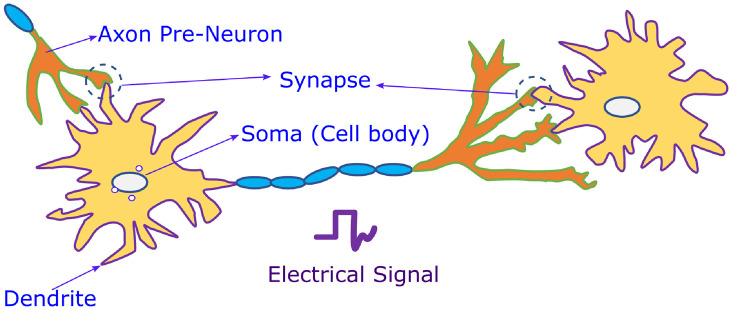
Biological neuron network.

**Fig 3:**
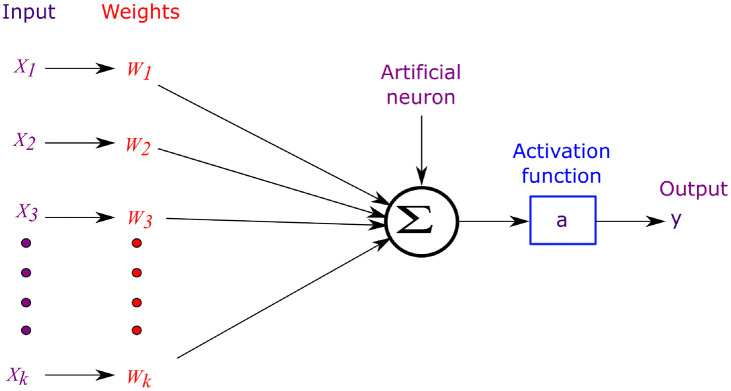
Structure of artificial neuron network.

Motivated by the biological structure, the neuron network is a basic structure comprising a block of neurons and a few synapses. It is illustrated in Fig 2 and mimics a biological neuron. It contains four components: synapses, axons, soma, and dendrites. The function of a synapse is to receive synaptic spikes from another neuron via its axon and transfer the information or impulse signal by the synaptic strength of that neuron [20]. Inspired by Biological Neural Network (BNN), the ANN structure is depicted in Fig 3. The neuron cell then uses its spiking pulse to perform spatio temporal integration and generates output spikes.

**Fig 4:**
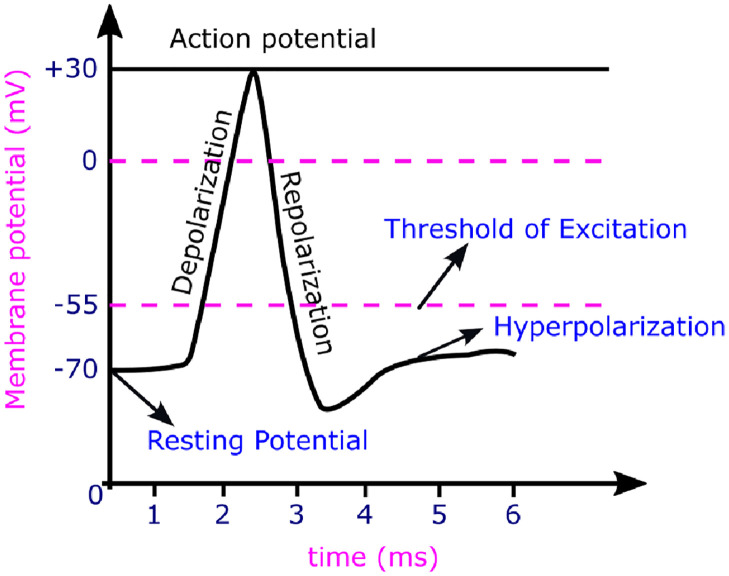
Action potential of the membrane.

Dendrites are used to collect and transmit signals to the soma. The Central Processing Unit (CPU), acts as the soma, and it performs the non-linear processing. The output signal must be produced when the input exceeds the threshold value. This is called the firing stage. The signals within the biological neuron are represented as the nerve pulses referred to as an action potential or spike, as shown in the Fig 4. Repolarisation and depolarization are related to the movement of potassium and sodium ions inside the cell. Following the firing, the neuron enters the refractory phase, during which it is less likely to fire. This process is known as firing and resting of biological neurons [21, 22].

## Memristor model

Depending on their retention duration, the memristors can be divided into two categories: volatile and non-volatile. Non-volatile memristive devices are an effective way to simulate the long-term plasticity observed in biology [23]. Thus, an ANN can be constructed using a crossbar array of nonvolatile memristive devices [24]. In contrast, volatile memristive devices can simulate short-term plasticity and exhibit nonlinearity and short-term memory [25].

Currently, memristive devices are used for synaptic devices to different human memory categories because the conductance change of memristive devices is similar to synaptic weight updates in the human brain. This indicates that the memristor is non-volatile since the state variable of the memristor can always recall its previous state, irrespective of the voltage value of the memristor at the moment of interruption [26].

**Fig 5:**
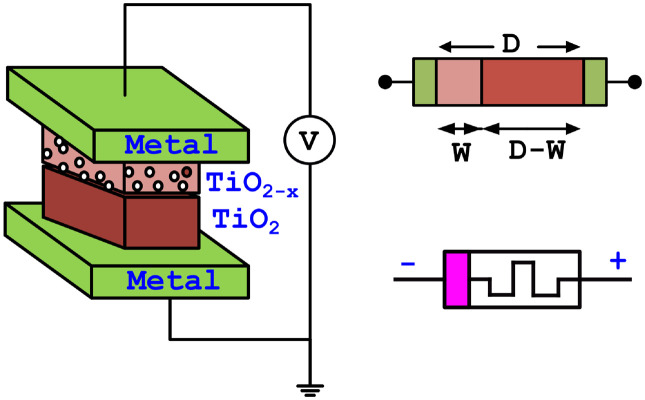
Memristor model and device symbol.

Leon Chua [27] formulated this memristor as the fourth fundamental element in 1971, first implemented in HP labs in 2008 [28]. A memristor can be expressed by magnetic flux and electric charge [22]. The device has been separated into two regions in the model as shown in Fig 5. The doped region with oxygen vacancies (*TiO*_2−*x*_) is one region can be represented by *W*. An additional region is the oxide region (*TiO*_2_), which is undoped and is designated as *D* − *W*.

The length of the memristor is indicated by *D*, and the size of the filled region can be expressed by *w*(*t*).

Comparing the conductance of the doped and oxide areas, the coated area has a higher conductance. When *w*(*t*) reaches *D*, or *w*(*t*) = *D*, the oxygen vacancies are enhanced by applying a positive bias and turning it into a low resistance state (*R*_*ON*_). When *w*(*t*) = 0, negative bias reduces the oxygen vacancies and causes them to transition into the high resistance state *R*_*OFF*_. As a result, memristors can be classified as either charge-controlled or flux-controlled devices [29]. A charge-controlled memristor exhibits the following behavior.

$$\begin{array}{c} M\left(q\right)=\frac{d\varphi (q)}{dq} \end{array}$$ (3)

$$\begin{array}{c} M\left(q\right)=\frac{\int v(t)dt}{\int i(t)dt}=\frac{v(t)}{i(t)} \end{array}$$ (4)

From Eqs 3 and 4, *M*(*q*) represents memristance, *v* is the designated memristor voltage, and *i* is the current.

I-V characteristics of a memristor given in Eq 5,

$$\begin{array}{c} v(t)=M(q(t))\cdot i(t) \end{array}$$ (5)

In terms of device parameters, memristance *M*(*q*) given in Eq 6.

$$\begin{array}{c} M\left(q\right)=R_{ON}\frac{w(t)}{D}+R_{OFF}(1-\frac{w(t)}{D}) \end{array}$$ (6)

Substitute Eq 6 in Eq 5, we get

$$\begin{array}{c} v\left(t\right)=[R_{ON}\frac{w(t)}{D}+R_{OFF}(1-\frac{w(t)}{D})]\times i\left(t\right) \end{array}$$ (7)

In Eq 7 *R*_*ON*_ and *R*_*OFF*_ are a memristor’s low and high resistances.

The memristor model is chosen based on the application requirements, accuracy, and suitability for the specific technology being used. Compared to other mathematical models that have been previously developed, the VTEAM (Voltage ThrEshold Adaptive Memristor) model has a well-established position in the literature and offers a reasonable level of accuracy. It also demonstrates flexibility, generality, and computational efficiency up to a certain threshold voltage. Kvatinsky et al. proposed the VTEAM model [30], which enables the memristor to electrically switch between the ON and OFF states with varying doping concentrations. The resistance of the memristor changes from one value to another to the threshold voltage. The resistance of the memristor adjusts in response to the applied voltage. When a positive threshold is reached, the device transitions from a high resistance state (HRS) to a low resistance state (LRS), and vice versa [31]. A voltage-controlled time-invariant memristive device that [29], VTEAM model is illustrated from Eqs 8 to 11.

$$\begin{array}{c} \frac{dw(t)}{dt}=\{\begin{array}{cc} k_{off}{\left(\frac{v(t)}{v_{off}}-1\right)}^{\alpha_{off}} & ;\;\;\;0<v_{off}<v\\ 0 & ;\;\;\;v_{on}<v<v_{off}\\ k_{on}{\left(\frac{v(t)}{v_{on}}-1\right)}^{\alpha_{on}} & ;\;\;\;v<v_{on}<0 \end{array} \end{array}$$ (8)

Linear dependence of the resistance and the state variable can be achieved, where the current–voltage relationship is,

$$\begin{array}{c} i\left(t\right)={\left[R_{on}+\frac{R_{off}-R_{on}}{w_{off}-w_{on}}\left(w-w_{on}\right)\right]}^{-1}\times v\left(t\right) \end{array}$$ (9)

$$\begin{array}{c} i\left(t\right)=\frac{e^{-\frac{\lambda }{w_{off}-w_{on}}\left(w-w_{on}\right)}}{R_{ON}}\times v\left(t\right) \end{array}$$ (10)

Here *k*_*off*_, *k*_*on*_, *α*_*off*_, and *α*_*on*_ are fitting constant variables. *v*_*on*_ and *v*_*off*_ are threshold voltages. The functions of *f*_*off*_(*w*) and *f*_*on*_(*w*) shows the derivative of the state variable depends on the state variable *w*, These functions operate similarly to window functions, limiting the state variable to *w* ∈ [*w*_*on*_, *w*_*off*_] boundaries. where λ denotes the normalizing factor,

$$\begin{array}{c} e^{\lambda }=(\frac{R_{off}}{R_{on}}) \end{array}$$ (11)

**Fig 6:**
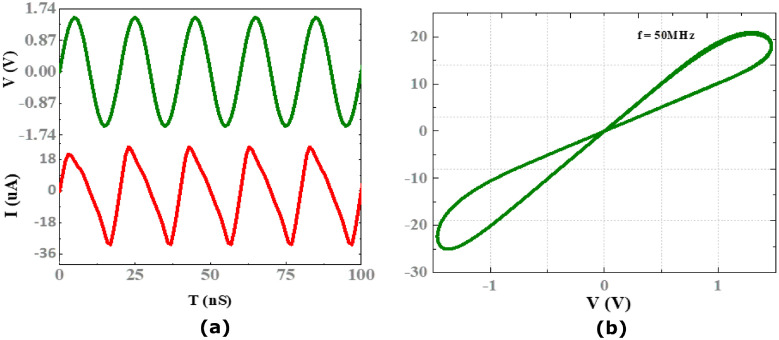
Memristor: (a) input, and output waveform; (b) hysteresis loop for frequency *f* = 50MHz.

Fig 6 displays the input and output waveforms, and the hysteresis loop of VTEAM memristor model. Table 1 lists the parameters of the memristor model that were used in the design.

**Table 1:** Memristor values.

Variable	Description	Value
*R*_*ON*_	Low Resistance state (ON)	100 Ω
*R*_*OFF*_	High Resistance state (OFF)	100 kΩ
*D*	Thickness of the memristor	3 nm
*μ*_*V*_	Mobility of ions	1e^−15 *m*^2^/*s*.*V*^
*f*(*w*)	Window function	0 < *w* < 1

## Artificial synapse

**Fig 7:**
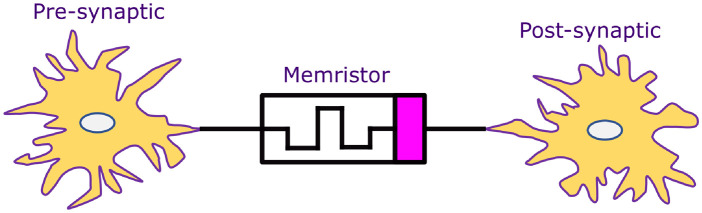
Memristor synapse.

Memristor-based artificial synapses have several benefits, including minimal power consumption, fast running speed (<1 ns), and synaptic weight updating [32]. The memristor synapse is illustrated in Fig 7. In biological systems, synapses are the bridges that transfer weighted spikes coming from presynaptic to postsynaptic neurons. The physical characteristics of neural synapses and ion migration-based operating mechanisms are strikingly similar to the electrical properties of a memristor. Compared to synapses made up of several transistors and capacitors, a single memristor can mimic the operation of neural synapses while consuming less power and area [33].

The ability of synapses to be controlled is a key factor in the transmission and exchange of electrical impulses between neurons. These electrical impulses have the potential to alter both the firing behavior of neurons and the dynamic properties of the ion channels within the neuron membrane. Exposure of media to electromagnetic fields causes polarization and magnetization, which alter brain activity and encode energy distribution [34].

Through the construction of neuromorphic hardware array systems, the synaptic devices in each array unit correspond to synapses in biological neural networks. These synaptic devices are required to mimic short-term and long-term plasticity for neuromorphic computing. Currently, memristive devices are among the candidates used for synaptic devices because the conductance change of memristive devices is similar to synaptic weight updates in the human brain [35].

### DT1M synapse

**Fig 8:**
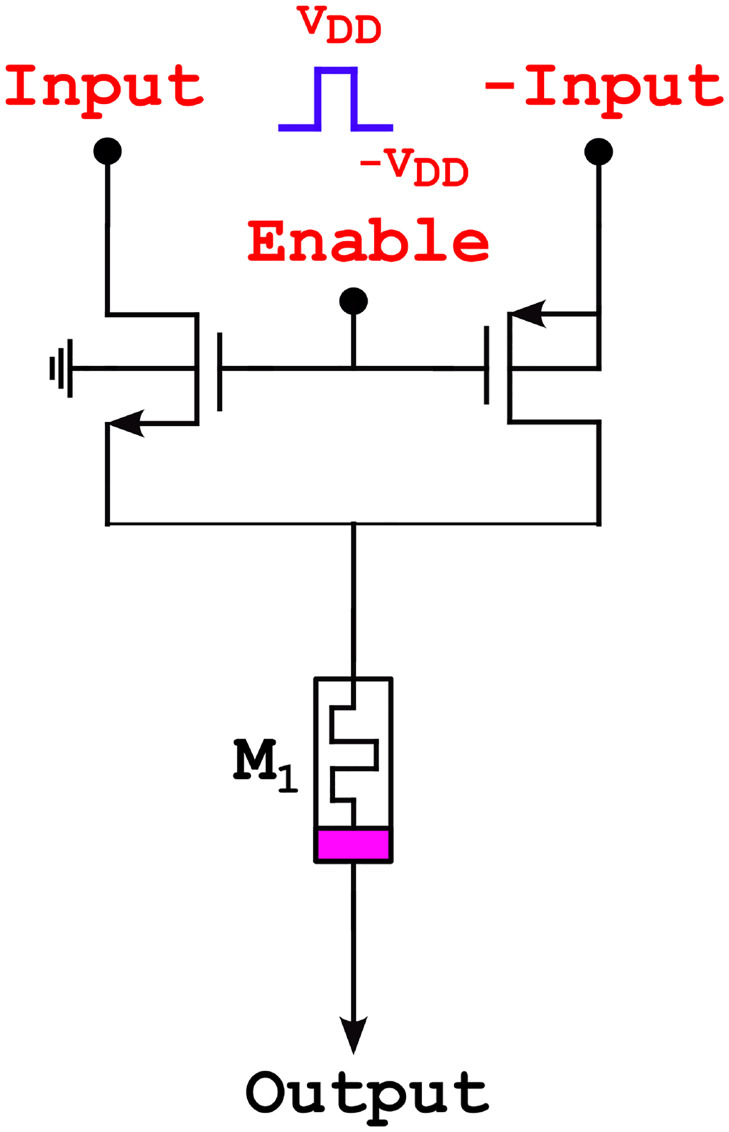
Memristor DT1M synapse.

The DT1M (Dual Transistor with One Memristor)synapse structure [36] is depicted in Fig 8, and it is linked with two inputs: a primary input and an inverted input of secondary signal. This structure allows control through the memristor synapse sign. The enable signal regulates both PMOS and NMOS device switching. The memristor value is updated, allowing the signal to control the current direction. The enable signal produces the positive and negative memristor weight signs.

The transmission gate transistors also functioned as the source and drain of these terminals according to the input. Additionally, Eq 12 provides the n-type transistor current (*I*) within the linear region.

$$\begin{array}{c} I=K((V_{\text{GS}}-V_{T})V_{\text{DS}}-\frac{1}{2}V_{\text{DS}}^{2}) \end{array}$$ (12)

The cutoff current flows (*I* = 0) when *V*_*GS*_ < *V*_*t*_. Similarly, Eq 13 provides the p-type MOSFET current within the linear region.

$$\begin{array}{c} I=-K((V_{\text{GS}}-V_{T})V_{\text{DS}}-\frac{1}{2}V_{\text{DS}}^{2}) \end{array}$$ (13)

The compact nature of the transistors used in synapses is a limitation of the design, while appropriate for small-scale problems, this can pose a serious issue for large-scale edge computing systems. As mentioned earlier, the representation of the circuit shows that the DT1M synapse operates at three voltage levels: *V*_*DD*_, −*V*_*DD*_, and 0*V*. The memristor’s voltage can be either positive or negative depending on the input. Where *V*_*T*_ is threshold voltage, and *K* is the conductance parameter of the transistor [37].

The following conditions are assumed for DT1M synapse is:

1. Both transistors are non-conducting (in the cutoff zone) if enable input *e* = 0. The output in this instance has zero voltage across the memristor, and the state variable remains unchanged.
2. In the linear region, the n-type transistor conducts whereas the p-type transistor is nonconducting if *e* = *V*_*DD*_.
3. In the linear region, the n-type transistor is nonconducting, and the p-type transistor is conducting if *e* = −*V*_*DD*_.

The comparison of the memristor’s conductivity, both transistors exhibit comparatively high conductivity when operating in the linear region.

The circuit’s two phases, reading and writing case functionality, are defined in Eqs 14 and 15. When it comes to reading performance,

$$\begin{array}{c} e\left(t\right)=\{\begin{array}{ccc} V_{DD} & ; & 0<t<0.5T_{rd}\\ -V_{DD} & ; & 0.5T_{rd}<t<0 \end{array} \end{array}$$ (14)

and writing performance,

$$\begin{array}{c} e\left(t\right)=\{\begin{array}{ccc} sign\left(y\right)\cdot V_{DD} & ; & 0<t-T_{rd}<b\left|y\right|\\ 0 & ; & b|y|<t<t_{wr} \end{array} \end{array}$$ (15)

### DTDM synapse

SNN’s synaptic weights are positive, negative, or zero. The synapse weights of DT1M artificial synapse consists only be positive and zero while DTDM artificial synapse contains positive, negative, and zero. These three synaptic weights (positive, negative, and zero) cannot be computed with a single memristor. Instead, a group of memristors is required to create a bipolarity weight.

**Fig 9:**
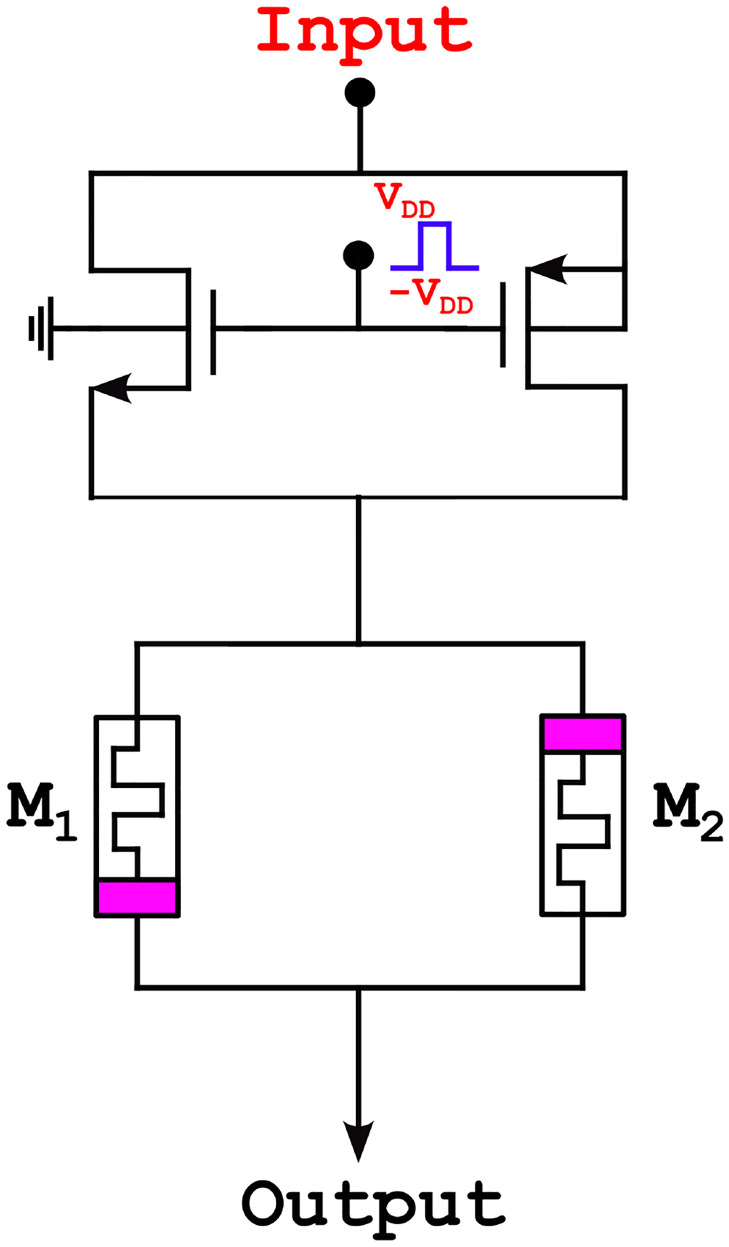
Memristor DTDM synapse.

To operate one memristor with a favorable current while passing another with an opposite current, memristors *M*_1_ and *M*_2_ are attached in the reverse directions. The configuration of this DTDM implementation [38], which involves two transistors and two memristor synapses are is depicted in Fig 9. Furthermore, currents are irrecoverable, and no weight is given to any applied spike input if both memristors have the same memristance value [39].

The memristance value of one memristor is altered by increasing or decreasing it, and the other memristor experiences a complementary value change that results in the weight change. Both memristors’ conductivity is proportionate to the synapse weight represent in Eq 16.

$$\begin{array}{c} G_{effec}=\frac{1}{M_{p}}-\frac{1}{M_{n}} \end{array}$$ (16)

## Memristor-based ML ANN with DTDM synapse circuit

### ML neuron

Researchers have investigated several neural structures and circuitry implementations. Several types of neuron models have been generated to mimic the electrical activity of neurons. The Leaky Integrate and Fire (LIF) and Morris Lecar(ML) approaches are most commonly employed in the literature [40, 41]. Despite this, the LIF neuron could not sufficiently represent ion transport kinetics through the membrane potential. As a result, using it is not bio-physically feasible. In most recent ML neuron implementations, sub-threshold semiconductors are used to reduce area and power [3].

To replicate the range of oscillatory behavior for calcium and potassium conductance in the muscle fiber of the giant barnacle, Catherine Morris and Harold Lecar developed the Morris-Lecar model (ML neuron model), which is based on a biological neuron model [42]. The proposed neuron network was designed with hybrid CMOS memristors and associated synapses to the neuron constructed with two transistors and two memristors. This neural network is based on the theoretical model approximation of Morris-Lecar, which has an advantage over LIF neurons in terms of power consumption.

When an external stimulus activates a neuron, it functions like an electrically charged particle whose electrical activity is altered. Therefore, it is crucial to use the model approach that takes these physical effects into account. Variations in magnetic or electric fields might therefore impact the electrically charged activity of neuron [34].

An alternative approach to SNN architecture known as “neuromorphic” forces the use of semiconducting fabrication methods, which might be connected to more sophisticated nano-scale devices such as memristors [43].

ML model Non-linear differential equations are shown in Eqs 17 and 18. From this equation, *C*_*m*_ is the membrane capacitance.

$$\begin{array}{c} C_{m}\frac{dv_{m}}{dt}=I_{ex}-G_{Ca}\cdot m_{ss}\left(v_{m}\right)-\left(v_{m}-E_{Ca}\right)-G_{k}\cdot n\left(v_{m}-E_{K}\right)-G_{L}\left(v_{m}-E_{L}\right) \end{array}$$ (17)

$$\begin{array}{c} \frac{dv}{dt}=\lambda \left(v_{m}\right)\left(n_{ss}\cdot \left(v_{m}\right)-n\right) \end{array}$$ (18)

Here, *I*_*ex*_ is an excitation synaptic current, *v*_*m*_ is a membrane potential. *E*_*K*_, *E*_*Ca*_, and *E*_*L*_ mentioned are the equilibrium ion potentials, and leakage conductance is signified in *G*_*Ca*_, *G*_*K*_, *G*_*L*_. The gating variables *m*_*ss*_ and *n*_*ss*_ at steady-state sodium and potassium formed as and written in Eqs 19 and 20.

$$\begin{array}{c} m_{ss}\left(v_{m}\right)=\frac{1}{2}[1+\text{tanh}\;(\frac{v_{m}-v_{1}}{v_{2}})] \end{array}$$ (19)

$$\begin{array}{c} n_{ss}\left(v_{m}\right)=\frac{1}{2}[1+\text{tanh}\;(\frac{v_{m}-v_{3}}{v_{4}})] \end{array}$$ (20)

$$\begin{array}{c} \lambda \left(v_{m}\right)=\lambda_{0}\;\text{cosh}\;(\frac{v_{m}-v_{3}}{2v_{4}}) \end{array}$$ (21)

Reference frequency can be acknowledged by λ_0_ from Eq 21. From this above equation, *v*_1_, *v*_2_, *v*_3_, and *v*_4_ are calcium activation potential and reciprocal slope respectively. The model parameter values of ML neuron model are listed in Table 2 [44].

**Table 2:** Model parameter values for ML neuron model.

Parameter	Name	Value
*E*_*Ca*_	Calcium equilibrium potential	1
*E*_*K*_	Potassium equilibrium potential	−0.7
*E*_*L*_	Leak equilibrium potential	−0.5
*G*_*Ca*_	Calcium ionic conductance	1
*G*_*K*_	Small ionic conductance	2
*G*_*L*_	Leak ionic conductance	0.5
*v*_1_	Calcium activation potential	−0.01
*v*_2_	Calcium reciprocal slope	−0.01
*v*_3_	Potassium activation potential	−0.01
*v*_4_	Potassium reciprocal slope	−0.01

**Fig 10:**
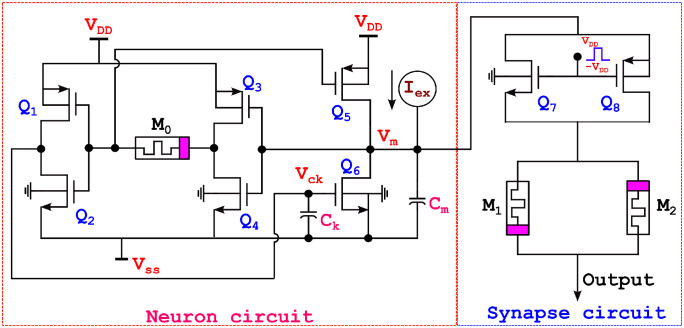
Proposed memristor based ML neuron network with DTDM.

Fig 10 illustrates the memristor-based ML neuron circuit with DTDM synapse. In the absence of excitation, the circuit is considered to be stable state and has a constant membrane voltage (*V*_*m*_).

### Circuit operation

The action potential in this instance is steady state voltage, and the neuron is expected to be at resting state potential. It indicates that the resting voltage is reduced to a level that is more compatible with the negative supply of the circuit. To minimize the voltage drop across the transistor *Q*_6_ terminal, the conductance of the transistor *Q*_6_ should be higher than that of the transistor *Q*_5_. The resting potential in a non-conducting state has been obtained by using Eq 22. Transistors *Q*_5_ and *Q*_6_ have some current flowing at the resting state and the membrane voltage is almost close to the negative supply.

$$\begin{array}{c} V_{rest}=\frac{G_{T_{5}}}{G_{T_{5}}+G_{T_{6}}}\times V_{DD} \end{array}$$ (22)

Even though membrane voltage (*V*_*m*_) remains constant when the excitation current (*I*_*ex*_) is insufficient to overcome the current that will be achieved in the transistor branch, the membrane voltage could be somewhat elevated in a higher stage rather than the initial resting voltage potential due to its limited compensation *Q*_5_. If the duration of this excitation is sufficient to reach the switching threshold of the first CMOS inverter, the output shows a significant increase during the positive feedback action of transistor *Q*_5_.

The inverters process the data, which are then stored in a memristor at *M*_0_ when the power is switched off. Because the memristor generates positive feedback, if the stimulation lasts long enough to reach the first inverter’s *Q*_3_ and *Q*_4_ switching thresholds the output will show a fast-rising. In this case, transistor *Q*_5_’s voltage (source to drain voltage) will decrease as it gets closer to the positive supply, hence reducing the rise in current as it passes through the transistor. Because of the exponential relationship between transistor *Q*_5_’s gate voltage and drain current, this occurs close to an increase.

If there is a positive voltage to the neuron, it will also increase. A larger membrane voltage reduces the potential drop across the source terminal and drain terminal of a transistor *Q*_5_. As a result, when the current flowing via transistor *Q*_5_ decreases, the rise of *V*_*m*_ will be effectively reduced. The capacitance *C*_*k*_ charges and the transistor’s drain current increases as *V*_*m*_ rises. When *V*_*m*_ reaches its maximum value, *Q*_5_ and *Q*_6_ dissipate the power. The presence of a memristor causes the neuron to be non-volatile. When the drain current of transistor *Q*_5_ rises, the membrane’s capacitance discharges, and *V*_*m*_ returns to its resting value. The membrane capacitance’s current charge accumulation determines its potential. The primary circuit characteristics that impact the dynamics and spike output is the inverters’ switching threshold, transistors’ conductivity margins, and the parasitic capacitance’s *C*_*m*_ and *C*_*k*_. Similarly if an excitation current (*I*_*ex*_) causes additional charge accumulation in the cell’s membrane capacitance. This robust amplification is brought about by the growing exponential change between the drain current and gate voltage at transistor *Q*_5_. The parasitic capacitances have influenced power consumption in the proposed neuron circuit. The power consumption varies based on the parasitic capacitance due to the dynamic supply current. The supply current is dominant in CMOS circuits because most of the power is consumed by moving charges in the parasitic capacitance in the CMOS gates.

The proposed neuron network with DTDM synapse circuit has memristors *M*_1_ and *M*_2_, such that *M*_1_ in the ON position, and *M*_2_ is OFF for a positive signal; in the opposite case, memristors *M*_1_ will be OFF, and *M*_2_ will be ON for a negative signal. When applying positive *V*_*dd*_ to enable the circuit, the *Q*_7_ transistor is ON condition, and the signal flows through memristors *M*_1_ and *M*_2_. The memristor *M*_1_ will be forward biased for positive signal, and memristor *M*_2_ will be reverse biased. The output follows through the memristor *M*_1_. For negative signal, memristor *M*_1_ will be reverse biased, and memrsitor *M*_2_ will be forward biased output flows through *M*_2_. Similarly, *Q*_7_ transistor will be OFF for applying negative *V*_*dd*_, and transistor *Q*_8_ will be ON and output flows through transistor *Q*_8_, and the same output flows through the memristor synapse based on the receiving signal. Finally, the neuron network output is obtained at the DTDM synapse. If the memristor’s memristance are equal, no current passes through the memristor; hence, neuron output is the same. The variations in the memristor memristance of the neuron network will cause the minimized output, it is shown in Table 3.

**Table 3:** Synaptic weight of memristor.

Memristor Condition	Variation in output
*M*1 = *M*2	101 mV
*M*1 > *M*2	90 mV
*M*1 < *M*2	95 mV

The circuit parameters are listed in Table 4. Excitation current *I*_*ex*_ corresponds to 20 pA, the supply voltage is 100 mV, and feedback capacitance and membrane capacitance’s are 8 fF and 12 fF, respectively, which can evaluate the efficacy of the proposed network.

**Table 4:** Parameter values for proposed neuron network circuit.

Transistors/ Capacitor	*Q*_1_	*Q*_2_	*Q*_3_	*Q*_4_	*Q*_5_	*Q*_6_	*Q*_7_	*Q*_8_	*C*_*m*_	*C*_*k*_
**Width (nm)**	360	120	360	120	300	120	360	120	12 fF	8 fF

Beyond their computational ability, neural networks and brain-inspired artificial neurons have found extensive applications in various fields, such as speech and image recognition, robotics, and healthcare [45, 46]. Using brain-inspired computing principles, scientists and engineers continue to expand the frontiers of artificial intelligence, propelling inventiveness and advancements in deep learning methods and cognitive systems [47–49]. Neuromorphic computing applications of artificial neural networks in various fields highlight their revolutionary influence on contemporary technology and society [32].

The performance metrics like spiking frequency, power consumption, energy, and maximum peak-to-peak voltage is calculated using the Cadence Virtuoso tool with 45nm CMOS technology is shown in Table 5.

**Table 5:** Performance metrics of proposed neuron network.

Neuron Network	Spike Freq.(Hz)	Power (W)	Energy (J/spike)	Max. Vp-p (mV)	Synaptic weight
Memristor based neuron with DTDM synapse	22.72k	12.55p	2.13f	101.18	Zero, Positive and Negative
Memristor based neuron with DT1M synapse	19.94k	13.23p	2.54f	121.24	Positive and Negative

We have compared the memristor based neuron network with the DTDM synapse and DT1M synapse. The performance metrics are improved in our proposed neuron network (with DTDM synaptic model) when compared to the DT1M synapse model. The results show that the DTDM synapse model has better metrics like spike frequency, power, and energy per spike when compared to existing models.

## Results and discussion

The results and analysis of the existing designs and the proposed neuron network with DTDM synapse circuits are discussed. In Cadence’s Virtuoso (Spectre simulator), a 45 nm technology node is used to evaluate the electrical behaviour and functionality of the proposed neuron network synapse models. Similarly, our proposed neuron models are developed using the same 45 nm technology as the memristor device’s behavioral model (Verilog-A code), which is invoked as a symbol in the Spectre simulator [50, 51].

**Fig 11:**
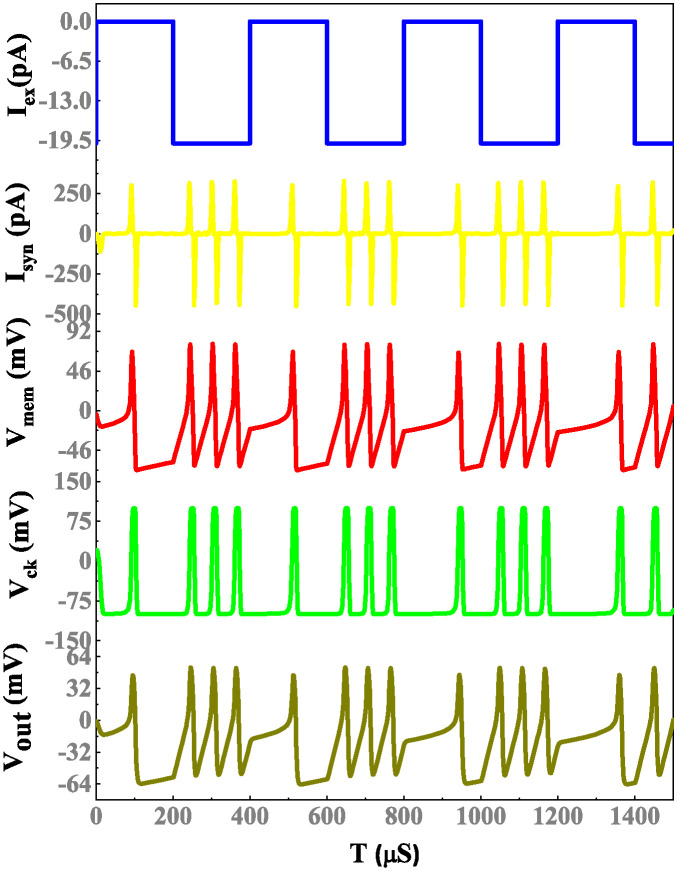
Proposed neuron network with DT1M synapse simulation results.

**Fig 12:**
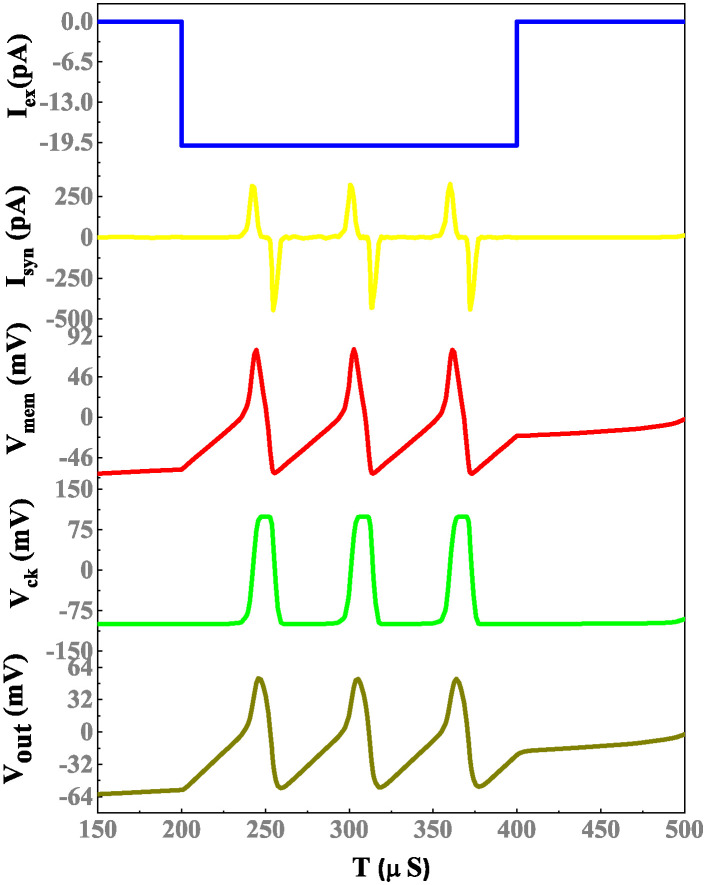
Proposed neuron network with DT1M synapse simulation results.

**Fig 13:**
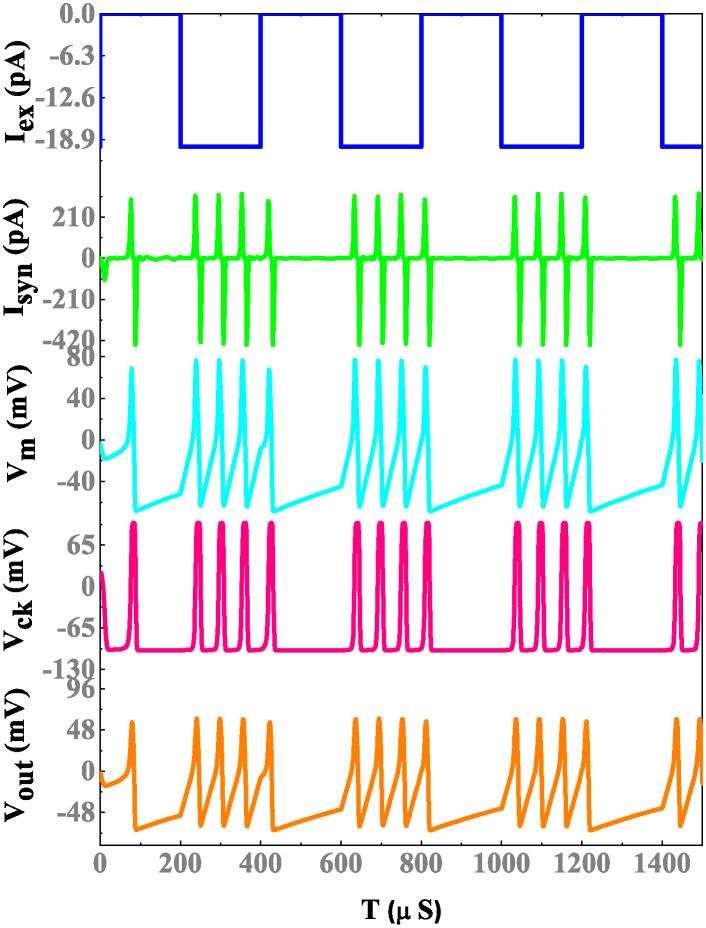
Proposed neuron network with DTDM synapse simulation results.

**Fig 14:**
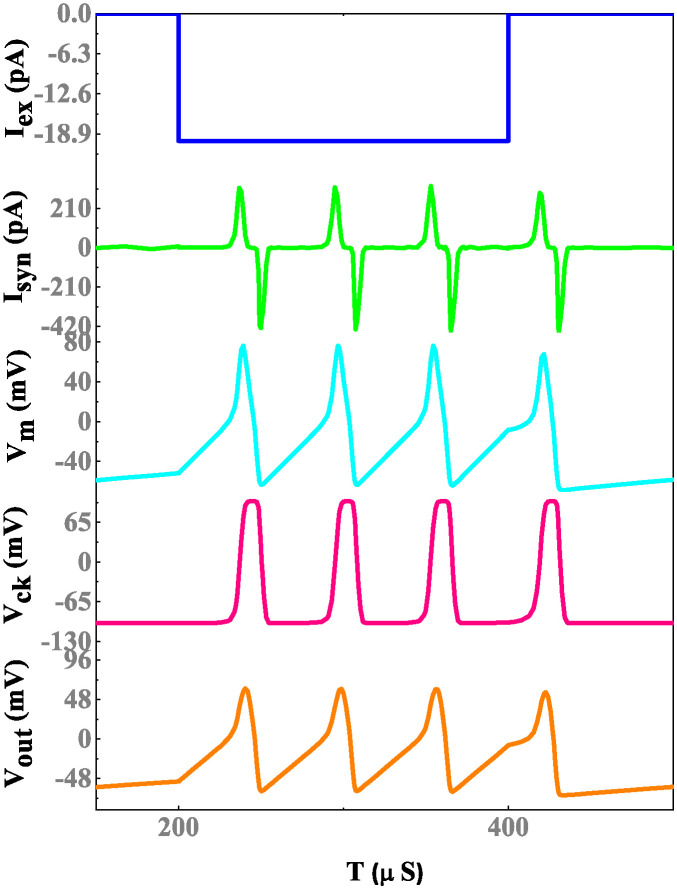
Proposed neuron network with DTDM synapse simulation results.

**Fig 15:**
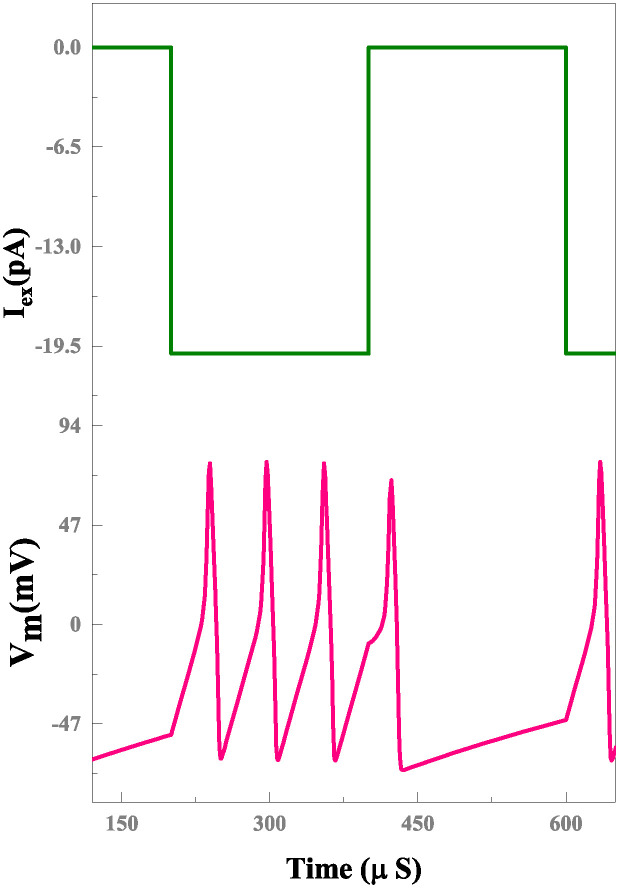
Proposed neuron network with DTDM synapse membrane voltage for *I*_*ex*_ = 20 pA.

**Fig 16:**
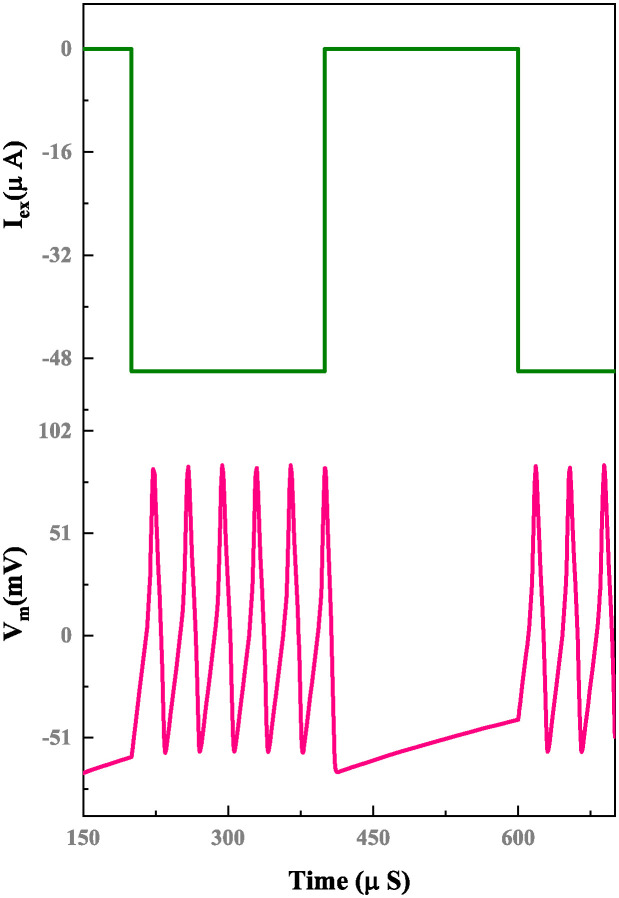
Proposed neuron network with DTDM synapse membrane voltage for *I*_*ex*_ = 60 pA.

The simulation results of a memristor device include an efficient evaluation of the memristor-based neuron network. The electrical properties of a proposed neuron network with DT1M simulation results are shown in Figs 11 and 12. For the proposed neuron network design, the circuit variables are *I*_*ex*_ which is excitation current, membrane potential (*V*_*m*_), current across the capacitance (*C*_*k*_) denoted as *I*_*ck*_ with their voltage being *V*_*ck*_. Finally, the synapse circuit output voltage is *V*_*out*_. Furthermore, a memristor-based neuron network with the DTDM synapse is illustrated in Figs 13 and 14, compared to the DT1M synapse network, DTDM synapse network enhances the synaptic properties. The proposed neuron network of membrane voltage wave forms are shown in Figs 15 and 16 with the excitation input currents (*I*_*ex*_) are 20 pA and 60 pA, respectively.

Table 6 shows that, the results of our proposed circuit design with the other comparable existing works, the results of our circuit shows that better performance, particularly regarding low power consumption. The proposed circuit provides a power consumption of 23.63 pW. Our design input supply voltage took only 100 mV, whereas 200 mV was used by I. Sourikopoulos et al [33]. In addition to the proposed design’s low power consumption, the other benefit is the circuit’s robustness. The proposed design achieved maximum spiking frequency and peak-to-peak voltage results. Compared to the current ML neuron with CMOS synapse [33], the suggested neuron network offered unrivaled performance with a 55% reduction in energy consumption. The suggested neuron network uses the less energy per spike and less power consumption compared to all other designs.

**Table 6:** Comparison between the proposed neuron with existing designs.

Parameters	This Work (a)	This Work (b)	I. Sourikopoulos et al. [33]	J.K. Han et al. [52]	K. Yue et al. [53]	Ronchini et al. [18]	Cruz-Albrecht et al. [54]	Moradi et al. [55]
Model	Memristor based neuron with DTDM synapse	Memristor based neuron with DT1M synapse	ML neuron with CMOS synapse	Artificial neuron	Simplified ML neuron	Izhikevich neuron	LIF neuron	LIF neuron
Technology node	45 nm	45 nm	65 nm	250 nm	65 nm	180 nm	90 nm	28 nm
Spike Freq. (Hz)	22.72k	19.94k	7.99k	11.7k	26k	NA	100	30
Power(W)	12.55p	13.23p	157.33p	1.5*μ*	105p	NA	40p	NA
Energy (J/spike)	2.13f	2.54f	4.75f	0.7p	4.0f	58.5f	0.4p	0.84p
Max. Vp-p(mV)	101.18	121.24	12.58	NA	112	NA	NA	NA

The limitations of our proposed neuron circuit provide better performance metrics by varying the excitation current from 0—100 pA. When the excitation current is above 100 pA, the neuron network will be saturated. The circuit operates at low power and is energy efficient when compared to other designs. Due to the memristor properties, our proposed neuron network has achieved low power consumption when compared with other designs.

Furthermore, the proposed network has a power consumption of 12.55 pW,which is less than that of compared to the CMOS neuron associated with CMOS synapse, which has a power consumption of 157.33 pW [33]. Moreover, compared to the CMOS-based ML neuron with CMOS synapse the increase in frequency is raised by 64.8%. The proposed design employs three memristors, two capacitors, and eight transistors to reduce the complexity of the memristor-based neuron network.

### Impact analysis of DC current, supply voltage, and temperature

**Fig 17:**
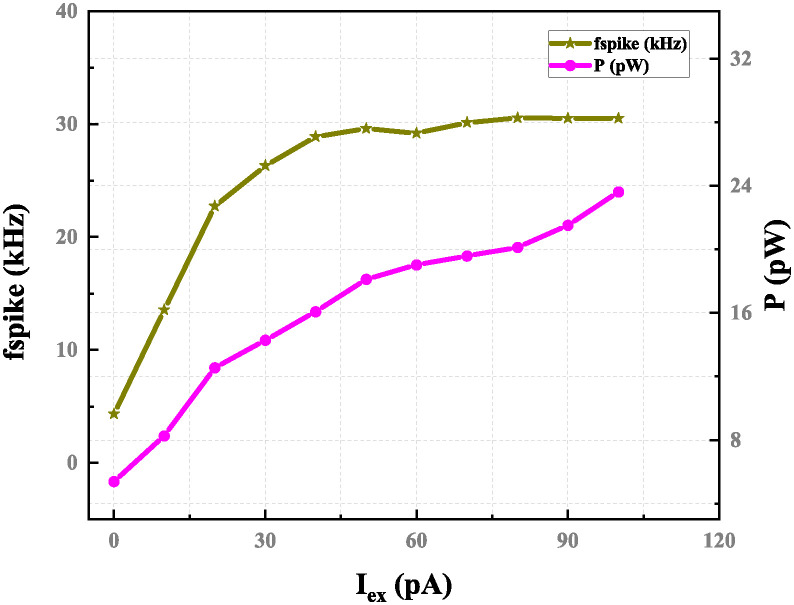
Spike frequency and power consumption versus excitation current with *V*_*DD*_=100 mv, *C*_*k*_=8 fF and *C*_*m*_=12 fF.

The proposed neuron network, excitation dc current range is displayed in Fig 17. The output spiking frequency increases from 4.42 kHz to 30.51 kHz when the input current varies from 10 pA to 100 pA. The reference voltage component uses total power consumption is 23.63 pW for the circuit performance, while the operating voltage is 100 mV.

**Fig 18:**
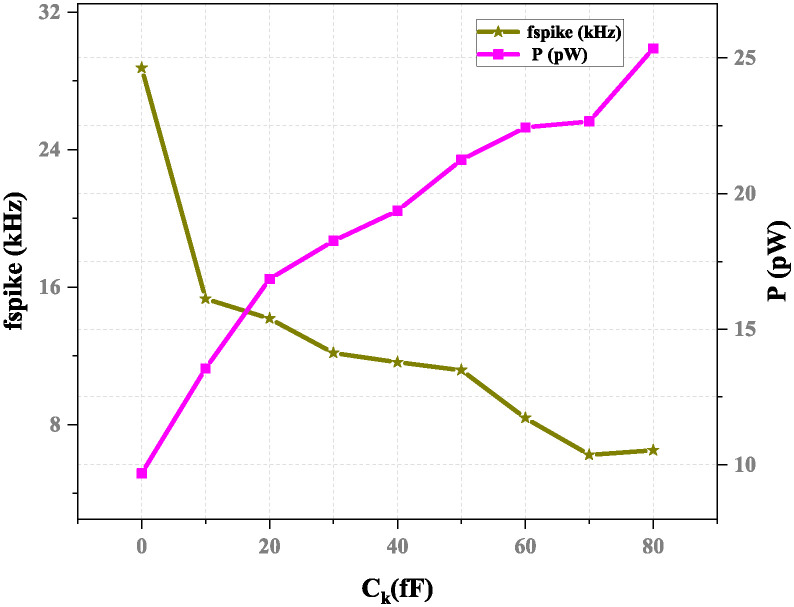
Spike frequency and power consumption versus capacitance *C*_*k*_ with *C*_*m*_= 25 fF and *I*_*ex*_= 20 pA.

If we consider the neuron circuit, we observe that the equivalent circuit of the output stage simultaneously consists of a conductance *G*_*m*_ and a capacitance *C*_*m*_. The noise voltage generated is represented by the root mean square (RMS) value, which is given by Eq 23. In the same manner, as the capacitor increases, the frequency decreases to 6.65 kHz is the minimum frequency achieved. Fig 18 represents the variation of *C*_*k*_ versus spike frequency. It shows that power consumption will increase as the spike frequency decreases when capacitance *C*_*k*_ increases.

$$\begin{array}{c} V_{m}=\sqrt{\frac{KT_{n}}{C_{m}}} \end{array}$$ (23)

The parasitic capacitance *C*_*k*_ and *C*_*m*_ have influenced power consumption in the proposed neuron circuit. The power consumption varies based on the parasitic capacitance due to the dynamic supply current. The supply current is dominant in CMOS circuits because most of the power is consumed by moving charges in the parasitic capacitance in the CMOS gates.

**Fig 19:**
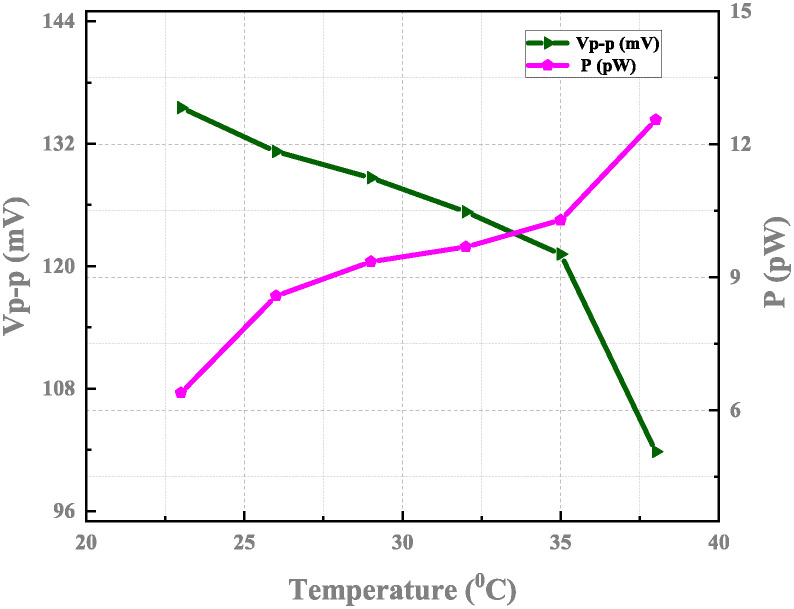
Output voltage Vp-p and power consumption versus temperature with *I*_*ex*_ = 20 pA, *C*_*k*_ = 8 fF, and *C*_*m*_ = 12 fF.

**Fig 20:**
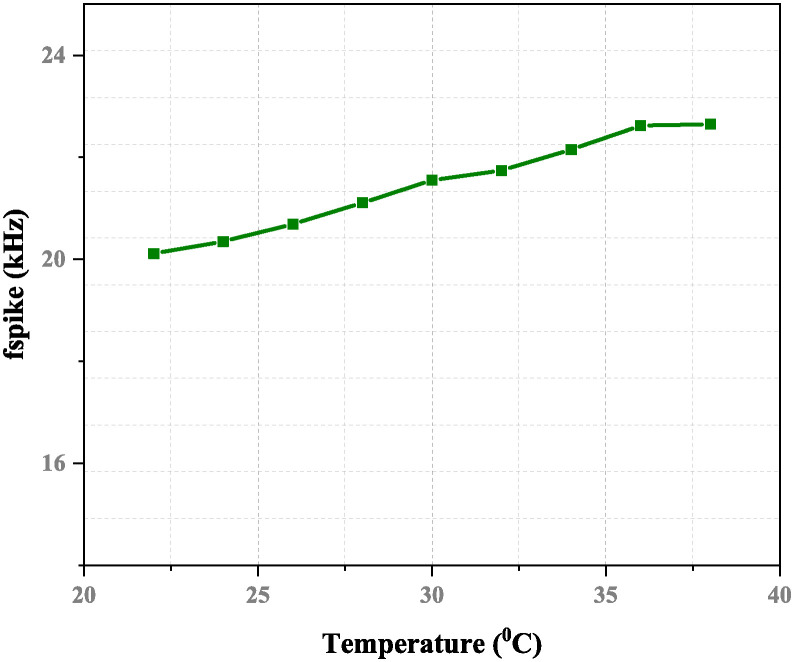
Spike frequency versus temperature with *I*_*ex*_ = 20 pA, *C*_*k*_ = 8 fF, and *C*_*m*_ = 12 fF.

The proposed neuron sub-threshold circuit’s response to ambient temperature has been calculated. Figs 19 and 20 depict the relationship between temperature versus voltage, power, and firing frequency. The impact on frequency variation tends to increase the temperature. The resultant spike frequency can vary within 1.3% when the temperature changes from 20°*C* to 40°*C* with a current as an input of 20 pA. When an input current can range from 0 to 150 pA, an artificial neuron network will produce spikes with a frequency between 4.32–30.56 kHz.

**Fig 21:**
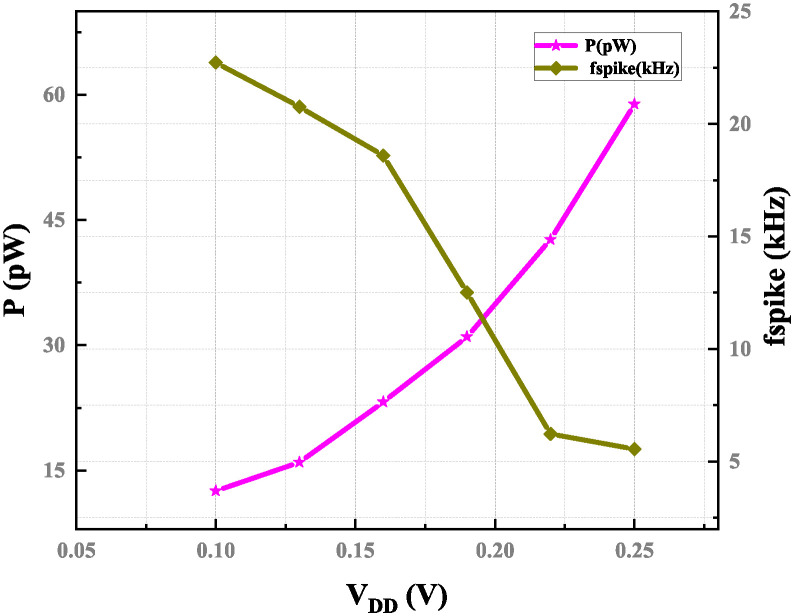
Power consumption and spike frequency versus supply voltage with *I*_*ex*_ = 20 pA, *C*_*k*_ = 8 fF, and *C*_*m*_ = 12 fF.

**Fig 22:**
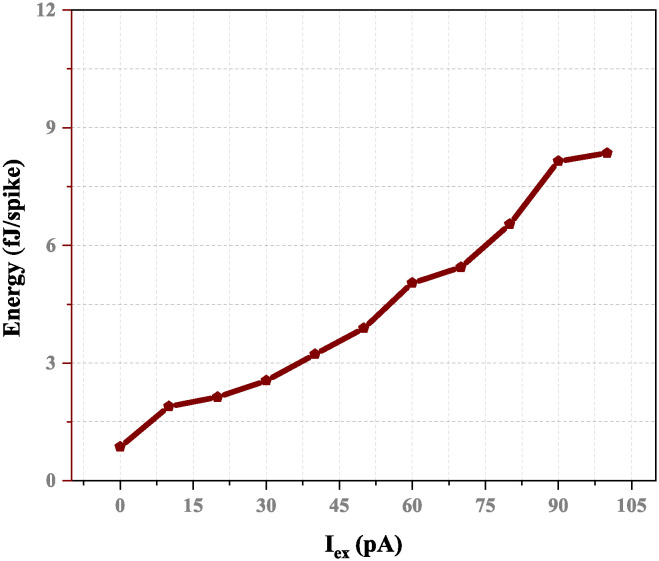
Energy (fJ/spike)versus excitation current with *V*_*DD*_=100 mv, *C*_*k*_=8 fF and *C*_*m*_=12 fF.

The proposed circuits are verified with the input current set to 20 pA and feedback capacitance of 8 fF to confirm the impact of ambient temperature and supply voltage *V*_*DD*_ on the working frequency. Fig 21 illustrates how the output frequency decreases as the power supply voltage, *V*_*DD*_, increases from 50 mV and 250 mV, resulting in an 11.66% variation in the maximum value output spike frequency. This circuit is resilient to a possible power supply and voltage noise and less affected by the actions of power supply ripples. Additionally, as the excitatory current increases, as seen in Fig 22, energy also increases 8.5 fJ of energy is the maximum with a supply voltage 100 mV.

Research Scientists have reproduced biological neurons’ spiking patterns and performance in the recommended circuits by enhancing their biological plausibility. The spiking frequency varies depending on the type of neuron network and specific tasks. Spike frequencies frequently communicate information to improve processing and response times in neural networks. In summary, the simulation results demonstrate that the proposed neuron network is efficient in connection with low power consumption and energy per spike, high spiking frequency, and maximum peak to peak voltage compared to various existing designs.

**Fig 23:**
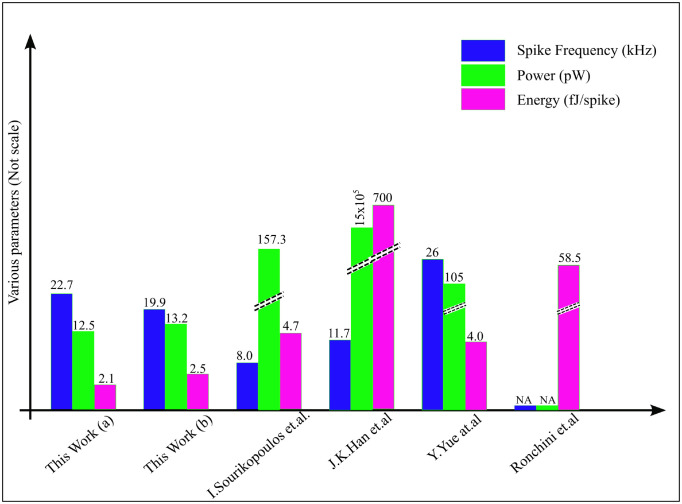
Comparison analysis for proposed neurons.

Fig 23 displays a comparison chart between the proposed neuron network with published works. Overall, based on comparing various neurons with the suggested circuit, the proposed circuit has an advantage of spiking frequency,energy per spike and power consumption. Izhikevich’s and LIF neurons’ spike frequency is shallow regarding Hz, and power consumption is high.

The main advantage of the proposed neuron network has low power consumption in nano-watts, reducing the complexity of the circuit, and efficient in energy per spike. Comparing our results to the existing neuron models, there is better performance. All things considered, the suggested circuit offers, the benefits of low power consumption, compatible energy per spike, frequency, and maximum peak-to-peak voltages are obtained.

The goal of the artificial neuron’s design is to function similarly to a biological neuron while enabling high-level integration. The important points were exposed by choosing to use the transistors through deep sub-threshold functioning from the standpoint of circuit design: the use of inverters to make the necessary non-linear functions easily achievable and the ability to operate with minimal supply voltage and associated capacitance, which allowed for significant energy consumption savings.

Achieving the gradient of current change encountered in biology mostly requires attaining frequency. A function of important circuit characteristics is used to express the output frequency in the second place. The neuron network produced by the ML model has biological significance, but it quickly became apparent that the resultant topology may be expanded to achieve a variety of performance optimization objectives. In this case, simplifying the design might reduce power consumption and energy.

## Conclusion

This paper proposes a memristor-based ML neuron network with a DTDM synapse model. The proposed circuits exist in its analog implementation of a memristor-based CMOS artificial neuron with DTDM, improving bi-polarity weights of synaptic characteristics. Furthermore, it achieves a maximum frequency of spiking at 30 kHz, low energy per spike, and low power consumption when compared with existing designs. The main feature of proposed design is its low power consumption. The simulated results demonstrate that the proposed neuron architecture holds promise for constructing energy efficient spiking neural networks implemented in neuromorphic computing applications.

## Supporting information

S1 Data (ZIP)
