# Supplementary material for: Low-power artificial neuron networks with enhanced synaptic functionality using dual transistor and dual memristor
Source: PLoS One. 2025 Jan 27;20(1):e0318009. doi: 10.1371/journal.pone.0318009 (PMC11771950; doi:10.1371/journal.pone.0318009)
Supplement: S1 Data — (ZIP) [file pone.0318009.s001.zip › Data_avaliabilty/1 .Memristor_model (Verilog-A code)/Memristor_model (Verilog-A code).pdf]

```
//////////////////////////////////////////////////
// VerilogA model for memristor                 //
//////////////////////////////////////////////////

`include "disciplines.vams"
`include "constants.h"

// define meter units for w parameter
nature distance
  access = Metr;
  units = "m";
  abstol = 0.01n;
endnature

discipline Distance
  potential distance;
enddiscipline


module Memristor(p, n,w_position);
  input p;//positive pin
  output n;//negative pin
  output w_position;// w-width pin

  electrical p, n,gnd;
  Distance w_position;
  ground gnd;

   parameter real model = 0;
// define the model:
// 0 - Linear Ion Drift;
// 1 - Simmons Tunnel Barrier;
// 2 - Team model;
// 3 - Nonlinear Ion Drift model
// 4 - Vteam model;

   parameter real window_type=0;
// define the window type:
// 0 - No window;
// 1 - Jogelkar window;
// 2 - Biolek window;
// 3 - Prodromakis window;
// 4 - Kvatinsky window (Team model only)
// 5 - Kvatinsky window2 (Vteam model only)

   parameter real dt=0;
// user must specify dt same as max step size in
// transient analysis & must be at least 3 orders
//smaller than T period of the source

   parameter real init_state=0.5;
// the initial state condition [0:1]
```

```
///////////// Linear Ion Drift model //////////////

 //parameters definitions and default values
    parameter real Roff = 200000;
    parameter real Ron = 100;
    parameter real D = 3n;
    parameter real uv = 1e-15;
    parameter real w_multiplied = 1e8;
// transformation factor for w/X width
// in meter units
    parameter real p_coeff = 2;
// Windowing function coefficient

parameter real J = 1;
// for prodromakis Window function


parameter real p_window_noise=1e-18;
// provoke the w width not to get stuck at
// 0 or D with p window

parameter real threshhold_voltage=0;

    // local variables
    real w;
    real dwdt;
    real w_last;
    real R;
    real sign_multply;
    real stp_multply;
    real first_iteration;


////////// Simmons Tunnel Barrier model ///////////

   //parameters definitions and default values
//for Simmons Tunnel Barrier model
    parameter real c_off = 3.5e-6;
    parameter real c_on = 40e-6;
    parameter real i_off = 115e-6;
    parameter real i_on = -8.9e-6;
    parameter real x_c = 107e-12;
    parameter real b = 500e-6;
    parameter real a_on = 2e-9;
    parameter real a_off = 1.2e-9;

    // local variables
    real x;
    real dxdt;
    real x_last;
```

```
/////////////////TEAM model////////////////////

    parameter real K_on=-8e-13;
    parameter real K_off=8e-13;
    parameter real Alpha_on=3;
    parameter real Alpha_off=3;
    parameter real v_on=-1.78;
    parameter real v_off=0.0115;
    parameter real IV_relation=0;
// IV_relation=0 means linear V=IR.
// IV_relation=1 means nonlinear V=I*exp{..}
    parameter real x_on=0;
    parameter real x_off=3e-09; // equals D

// local variables
    real lambda;


/////////////Nonlinear Ion Drift model /////////////

    parameter real alpha = 2;
    parameter real beta  = 9;
    parameter real c     = 0.01;
    parameter real g     = 4;
    parameter real N     = 14;
    parameter real q     = 13;
    parameter real a     = 4;

 analog function integer sign;
//Sign function for Constant edge cases
     real arg; input arg;
     sign = (arg >= 0 ? 1 : -1 );
 endfunction

 analog function integer stp;        //Stp function
     real arg; input arg;
     stp = (arg >= 0 ? 1 : 0 );
 endfunction





/////////////////// MAIN ///////////////////////

 analog begin

    if(first_iteration==0) begin
        w_last=init_state*D;
```

```
// if this is the first iteration,
//start with w_init
        x_last=init_state*D;
// if this is the first iteration,
// start with x_init
    end

/////////////Linear Ion Drift model //////////////

if (model==0) begin // Linear Ion Drift model

    dwdt =(uv*Ron/D)*I(p,n);

     //change the w width only if the
 // threshhold_voltage permits!
       if(abs(I(p,n))<threshhold_voltage/R) begin
            w=w_last;
        dwdt=0;
       end

// No window
    if ((window_type==0)|| (window_type==4)) begin

        w=dwdt*dt+w_last;

    end // No window

// Jogelkar window
     if (window_type==1) begin

        if (sign(I(p,n))==1) begin
            sign_multply=0;
            if(w<p_window_noise) begin
            sign_multply=1;
            end
        end
        if (sign(I(p,n))==-1) begin
                sign_multply=0;
                if(w>(D-p_window_noise)) begin
                    sign_multply=-1;
                end
        end

        w=dwdt*dt*(1-pow(pow(2*w/D-
1,2),p_coeff))+w_last+sign_multply*p_window_noise;

    end // Jogelkar window


// Biolek window
    if (window_type==2) begin
```

```
        if (stp(-I(p,n))==1) begin
            stp_multply=1;
        end
        if (stp(-I(p,n))==0) begin
                stp_multply=0;
                end

       w=dwdt*dt*(1-pow(pow(w/D-stp_multply,2),p_coeff))+w_last;

    end // Biolek window


  // Prodromakis window
    if (window_type==3) begin

        if (sign(I(p,n))==1) begin
            sign_multply=0;
            if(w<p_window_noise) begin
            sign_multply=1;
            end
        end
        if (sign(I(p,n))==-1) begin
                sign_multply=0;
                if(w>(D-p_window_noise)) begin
                    sign_multply=-1;
                    end
        end

        w=dwdt*dt*J*(1-pow(pow(w/D-
0.5,2)+0.75,p_coeff))+w_last+sign_multply*p_window_noise;

    end  // Prodromakis window

    if (w>=D) begin
        w=D;
                dwdt=0;
    end

    if (w<=0) begin
        w=0;
                dwdt=0;
    end


       //update the output ports(pins)
       R=Ron*w/D+Roff*(1-w/D);
       w_last=w;
       Metr(w_position) <+ w*w_multiplied;
       V(p,n) <+ (Ron*w/D+Roff*(1-w/D))*I(p,n);
       first_iteration=1;

end // end Linear Ion Drift model
```

```
///////// Simmons Tunnel Barrier model ///////////


if (model==1) begin // Simmons Tunnel Barrier model

    if (sign(I(p,n))==1) begin

       dxdt =c_off*sinh(I(p,n)/i_off)*exp(-exp((x_last-a_off)/x_c-
abs(I(p,n)/b))-x_last/x_c);
    end


    if (sign(I(p,n))==-1) begin
       dxdt =c_on*sinh(I(p,n)/i_on)*exp(-exp((a_on-x_last)/x_c-
abs(I(p,n)/b))-x_last/x_c);

    end


        x=x_last+dt*dxdt;


     if (x>=D) begin
            x=D;
            dxdt=0;
     end
     if (x<=0) begin
            x=0;
            dxdt=0;
     end


       //update the output ports(pins)
       R=Ron*(1-x/D)+Roff*x/D;
       x_last=x;
       Metr(w_position) <+ x/D;
       V(p,n) <+ (Ron*(1-x/D)+Roff*x/D)*I(p,n);
       first_iteration=1;

end // end Simmons Tunnel Barrier model



///////////////////// TEAM model /////////////////


if (model==2) begin // TEAM model

    if (I(p,n) >= i_off) begin
        dxdt =K_off*pow((I(p,n)/i_off-1),Alpha_off);
```

```
    end

    if (I(p,n) <= i_on) begin
        dxdt =K_on*pow((I(p,n)/i_on-1),Alpha_on);
    end

        if ((i_on<I(p,n)) && (I(p,n)<i_off)) begin
    dxdt=0;
    end


// No window
    if (window_type==0) begin

    x=x_last+dt*dxdt;

    end // No window

    // Jogelkar window
if (window_type==1) begin

     if (sign(I(p,n))==1) begin
     sign_multply=0;
     if(x<p_window_noise) begin
          sign_multply=1;
     end
    end
    if (sign(I(p,n))==-1) begin
     sign_multply=0;
     if(x>(D-p_window_noise)) begin
          sign_multply=-1;
     end
     end

        x=x_last+dt*dxdt*(1-pow(pow((2*x_last/D-
1),2),p_coeff))+sign_multply*p_window_noise;

    end // Jogelkar window


// Biolek window
    if (window_type==2) begin

        if (stp(-I(p,n))==1) begin
            stp_multply=1;
        end
        if (stp(-I(p,n))==0) begin
            stp_multply=0;
            end

        x=x_last+dt*dxdt*(1-pow(pow((x_last/D-
stp_multply),2),p_coeff));
```

```
    end  // Biolek window


// Prodromakis window
    if (window_type==3) begin

      if (sign(I(p,n))==1) begin
      sign_multply=0;
      if(x<p_window_noise) begin
           sign_multply=1;
      end
    end
    if (sign(I(p,n))==-1) begin
      sign_multply=0;
      if(x>(D-p_window_noise)) begin
           sign_multply=-1;
      end
      end
            x=x_last+dt*dxdt*J*(1-pow((pow((x_last/D-
0.5),2)+0.75),p_coeff))+sign_multply*p_window_noise;

    end  // Prodromakis window


//Kvatinsky window
    if (window_type==4) begin

           if (I(p,n) >= 0) begin
           x=x_last+dt*dxdt*exp(-exp((x_last-a_off)/x_c));
           end

       if (I(p,n) < 0) begin
           x=x_last+dt*dxdt*exp(-exp((a_on-x_last)/x_c));
       end

        end // Kvatinsky window

    if (x>=D) begin
    dxdt=0;
    x=D;
    end

    if (x<=0) begin
    dxdt=0;
    x=0;
    end

       lambda = ln(Roff/Ron);

     //update the output ports(pins)
        x_last=x;
```

```
        Metr(w_position) <+ x/D;

     if (IV_relation==1) begin

        V(p,n) <+ Ron*I(p,n)*exp(lambda*(x-x_on)/(x_off-x_on));

     end

     else if (IV_relation==0) begin

        V(p,n) <+ (Roff*x/D+Ron*(1-x/D))*I(p,n);

     end

    first_iteration=1;

end // end Team model


//////////// Nonlinear Ion Drift model ////////////


if (model==3) begin // Nonlinear Ion Drift model

    if (first_iteration==0) begin
        w_last=init_state;
    end

    dwdt = a*pow(V(p,n),q);


// No window
    if ((window_type==0) || (window_type==4)) begin
        w=w_last+dt*dwdt;
        end // No window


  // Jogelkar window
  if (window_type==1) begin

      if (sign(I(p,n))==1) begin
      sign_multply=0;
      if(w<p_window_noise) begin
           sign_multply=1;
      end
    end
    if (sign(I(p,n))==-1) begin
      sign_multply=0;
      if(w>(D-p_window_noise)) begin
           sign_multply=-1;
      end
      end
```

```
        w=w_last+dt*dwdt*(1-pow(pow((2*w_last-
1),2),p_coeff))+sign_multply*p_window_noise;

    end // Jogelkar window


// Biolek window
    if (window_type==2) begin

        if (stp(-V(p,n))==1) begin
            stp_multply=1;
        end
        if (stp(-V(p,n))==0) begin
                stp_multply=0;
            end

        w=w_last+dt*dwdt*(1-pow(pow((w_last-stp_multply),2),p_coeff));

    end // Biolek window


// Prodromakis window
    if (window_type==3) begin

      if (sign(I(p,n))==1) begin
      sign_multply=0;
      if(w<p_window_noise) begin
           sign_multply=1;
      end
    end
    if (sign(I(p,n))==-1) begin
      sign_multply=0;
      if(w>(D-p_window_noise)) begin
           sign_multply=-1;
      end
      end

            w=w_last+dt*dwdt*J*(1-pow((pow((w_last-
0.5),2)+0.75),p_coeff))+sign_multply*p_window_noise;

    end  // Prodromakis window

    if (w>=1) begin
        w=1;
        dwdt=0;
    end

    if (w<=0) begin
        w=0;
        dwdt=0;
    end
```

```
    //change the w width only if the
// threshhold_voltage permits!

        if(abs(V(p,n))<threshhold_voltage) begin
            w=w_last;
        end

      //update the output ports(pins)
       w_last=w;
           Metr(w_position) <+ w;
           I(p,n) <+
pow(w,N)*beta*sinh(alpha*V(p,n))+c*(exp(g*V(p,n))-1);
           first_iteration=1;

end // end Nonlinear Ion Drift model

///////////////////// VTEAM model //////////////////


if (model==4) begin // VTEAM model

    if (V(p,n) >= v_off) begin
        dxdt =K_off*pow((V(p,n)/v_off-1),Alpha_off);
    end

    if (V(p,n) <= v_on) begin
        dxdt =K_on*pow((V(p,n)/v_on-1),Alpha_on);
    end

        if ((v_on<V(p,n)) && (V(p,n)<v_off)) begin
    dxdt=0;
    end


// No window
    if (window_type==0) begin

    x=x_last+dt*dxdt;

    end // No window

    // Jogelkar window
if (window_type==1) begin

     if (sign(V(p,n))==1) begin
     sign_multply=0;
     if(x<p_window_noise) begin
           sign_multply=1;
     end
    end
    if (sign(V(p,n))==-1) begin
```

```
      sign_multply=0;
      if(x>(D-p_window_noise)) begin
           sign_multply=-1;
      end
      end


         x=x_last+dt*dxdt*(1-pow(pow((2*x_last/D-
1),2),p_coeff))+sign_multply*p_window_noise;

    end // Jogelkar window


// Biolek window
    if (window_type==2) begin

        if (stp(-V(p,n))==1) begin
            stp_multply=1;
        end
        if (stp(-V(p,n))==0) begin
            stp_multply=0;
            end

        x=x_last+dt*dxdt*(1-pow(pow((x_last/D-
stp_multply),2),p_coeff));

    end  // Biolek window


// Prodromakis window
    if (window_type==3) begin


      if (sign(V(p,n))==1) begin
      sign_multply=0;
      if(x<p_window_noise) begin
           sign_multply=1;
      end
    end
    if (sign(V(p,n))==-1) begin
      sign_multply=0;
      if(x>(D-p_window_noise)) begin
           sign_multply=-1;
      end
      end

            x=x_last+dt*dxdt*J*(1-pow((pow((x_last/D-
0.5),2)+0.75),p_coeff))+sign_multply*p_window_noise;

    end  // Prodromakis window
```

```
//Kvatinsky window2 VTEAM only
    if (window_type==5) begin

           if (V(p,n) >= 0) begin
           x=x_last+dt*dxdt*exp(-exp((x_last-a_off)/x_c));
           end

       if (V(p,n) < 0) begin
           x=x_last+dt*dxdt*exp(-exp((a_on-x_last)/x_c));
       end

        end // Kvatinsky window

    if (x>=D) begin
    dxdt=0;
    x=D;
    end

    if (x<=0) begin
    dxdt=0;
    x=0;
    end

       lambda = ln(Roff/Ron);

     //update the output ports(pins)
        x_last=x;
        Metr(w_position) <+ x/D;

     if (IV_relation==1) begin

        V(p,n) <+ Ron*I(p,n)*exp(lambda*(x-x_on)/(x_off-x_on));

     end

     else if (IV_relation==0) begin

        V(p,n) <+ (Roff*x/D+Ron*(1-x/D))*I(p,n);

     end

    first_iteration=1;

end // end VTEAM model


  end   // end analog

endmodule
```
