# Supplementary material for: Low-power artificial neuron networks with enhanced synaptic functionality using dual transistor and dual memristor
Source: PLoS One. 2025 Jan 27;20(1):e0318009. doi: 10.1371/journal.pone.0318009 (PMC11771950; doi:10.1371/journal.pone.0318009)
Supplement: S1 Data — (ZIP) [file pone.0318009.s001.zip › Data_avaliabilty/1 .Memristor_model (Verilog-A code)/Memristor_values.pdf]

You have to press Q to enter values of memristor, amplitude =1.5V, frequency= 2kHz

Parameter	Values
Model	4
Window type	0
Dt	1e-06
Initial_data	0.5<br>0 for LRS<br>1 for HRS
Roff	100k ohm
Ron	100 ohm
D	3e-09
μ V	1e-15
W_multiplied	1e+09
P_coeff	2
J	1.5
P_window_noise	1e-18
Threshold voltage	0
C_off	3.5e-06
C_on	4e-05
I_off	0.000115
I_on	-8.9e-06
X_c	107e-11
B	0.0005
A_on	3e-09
A_off	0
K_on	-1
K_off	1
Alpha_on	1
Alpha_off	1
V_on	-1
V_off	1
IV relation	0
X_on	0
X_off	3e-09
Alpha	2
Beta	9
C	0.01
G	4
N	14
Q	13
A	4
