# Supplementary figures and images for: Low-power artificial neuron networks with enhanced synaptic functionality using dual transistor and dual memristor
Source: PLoS One. 2025 Jan 27;20(1):e0318009. doi: 10.1371/journal.pone.0318009 (PMC11771950; doi:10.1371/journal.pone.0318009)

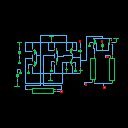

Supplement: S1 Data — (ZIP) [file pone.0318009.s001.zip › Data_avaliabilty/2. Cadence_Design files/mema2t2m/schematic/thumbnail_128x128.png]

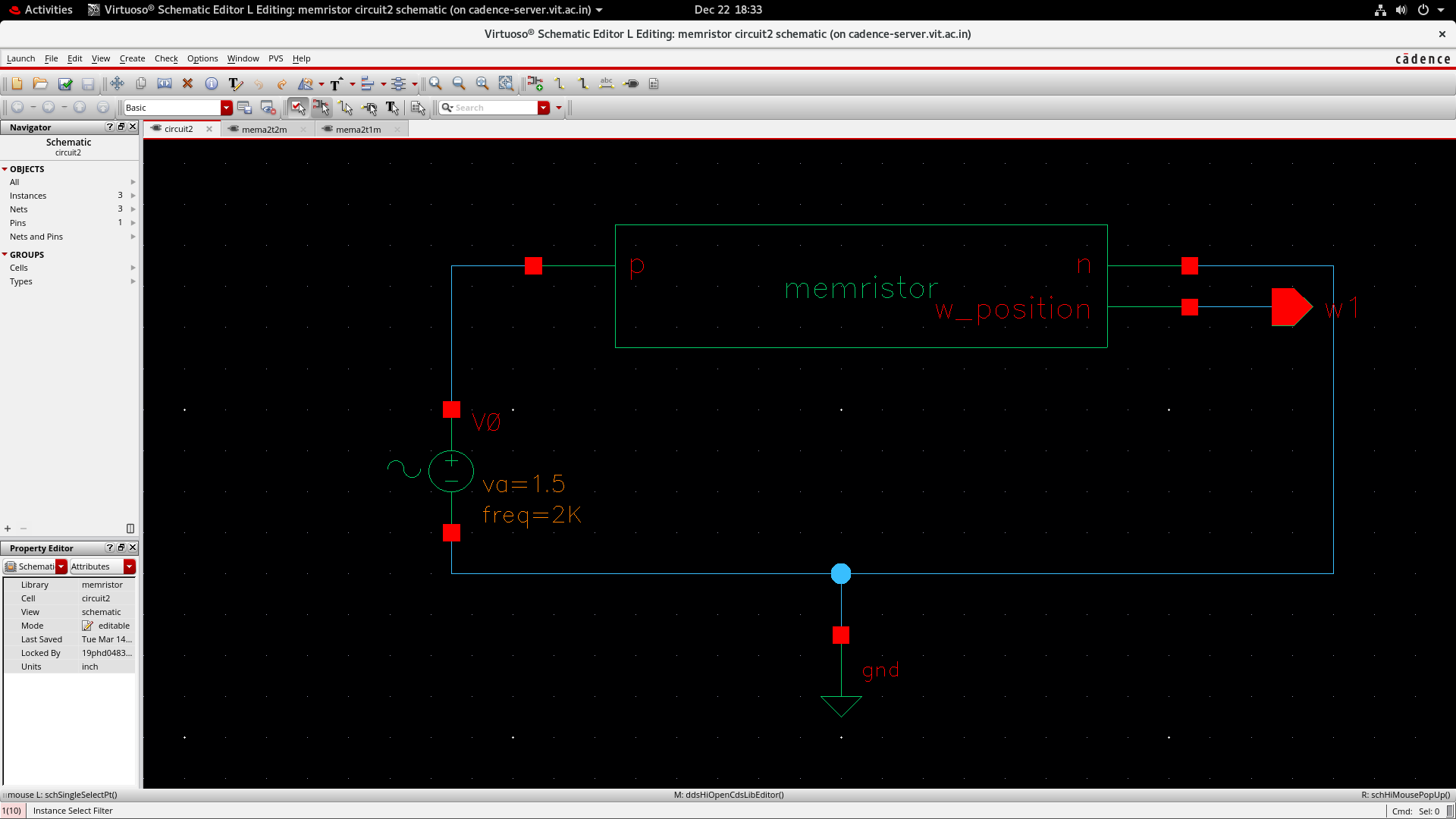

Supplement: S1 Data — (ZIP) [file pone.0318009.s001.zip › Data_avaliabilty/3. Screenshots of cirucits_and_results/memristor.png]

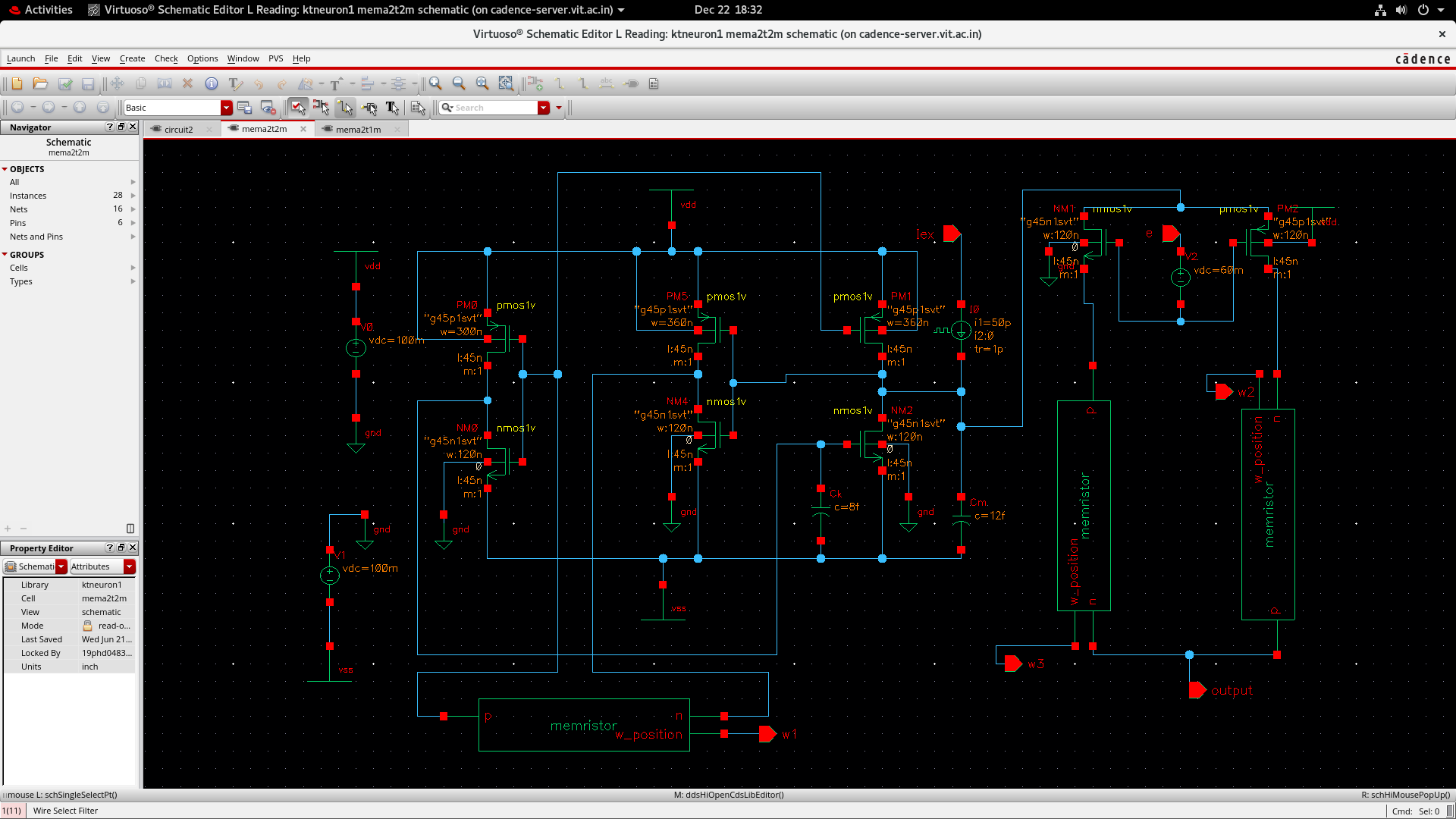

Supplement: S1 Data — (ZIP) [file pone.0318009.s001.zip › Data_avaliabilty/3. Screenshots of cirucits_and_results/ML neuron network with DTDM.png]

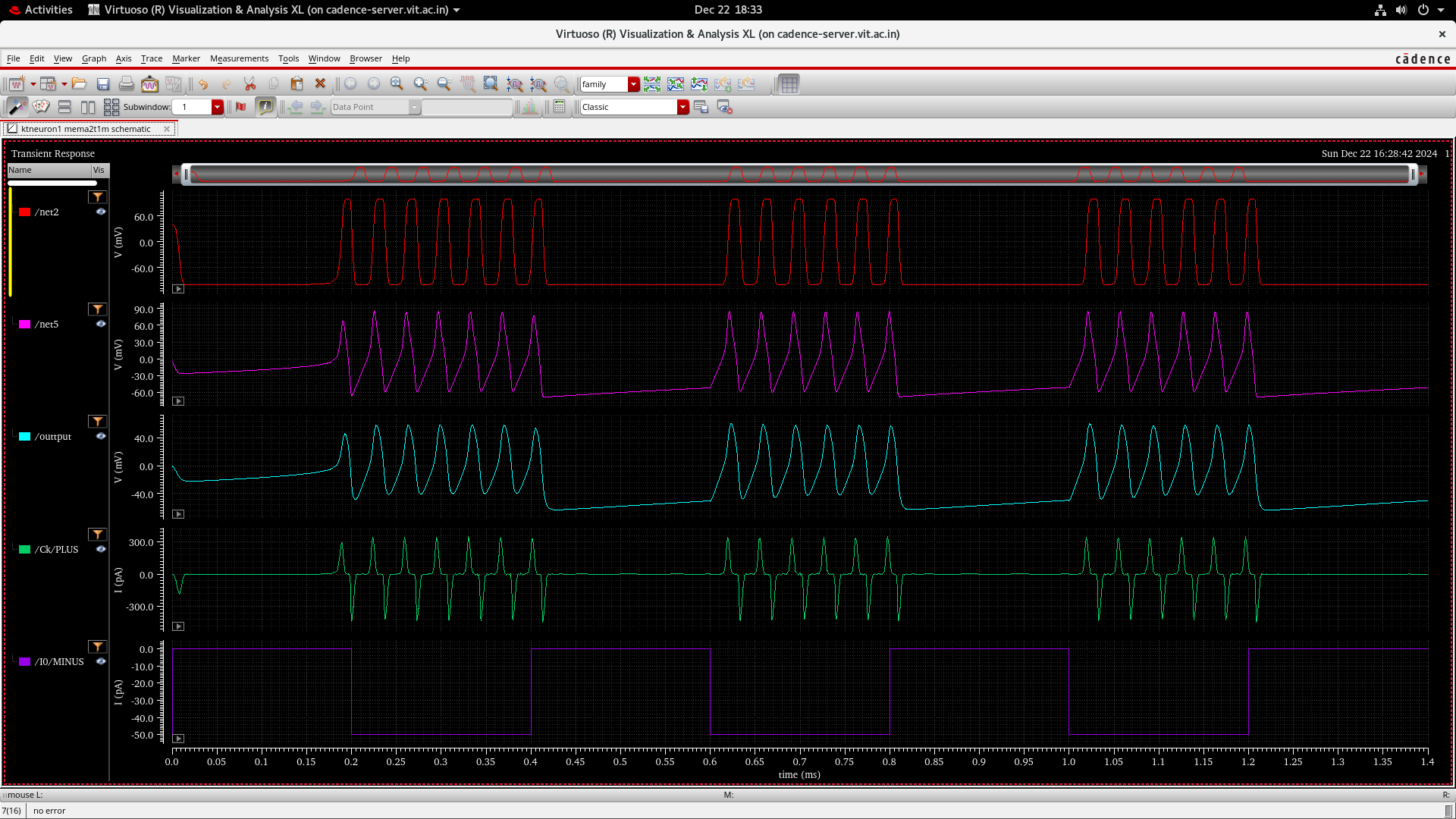

Supplement: S1 Data — (ZIP) [file pone.0318009.s001.zip › Data_avaliabilty/3. Screenshots of cirucits_and_results/Output_waveform_ML neuron with DT1M.png]

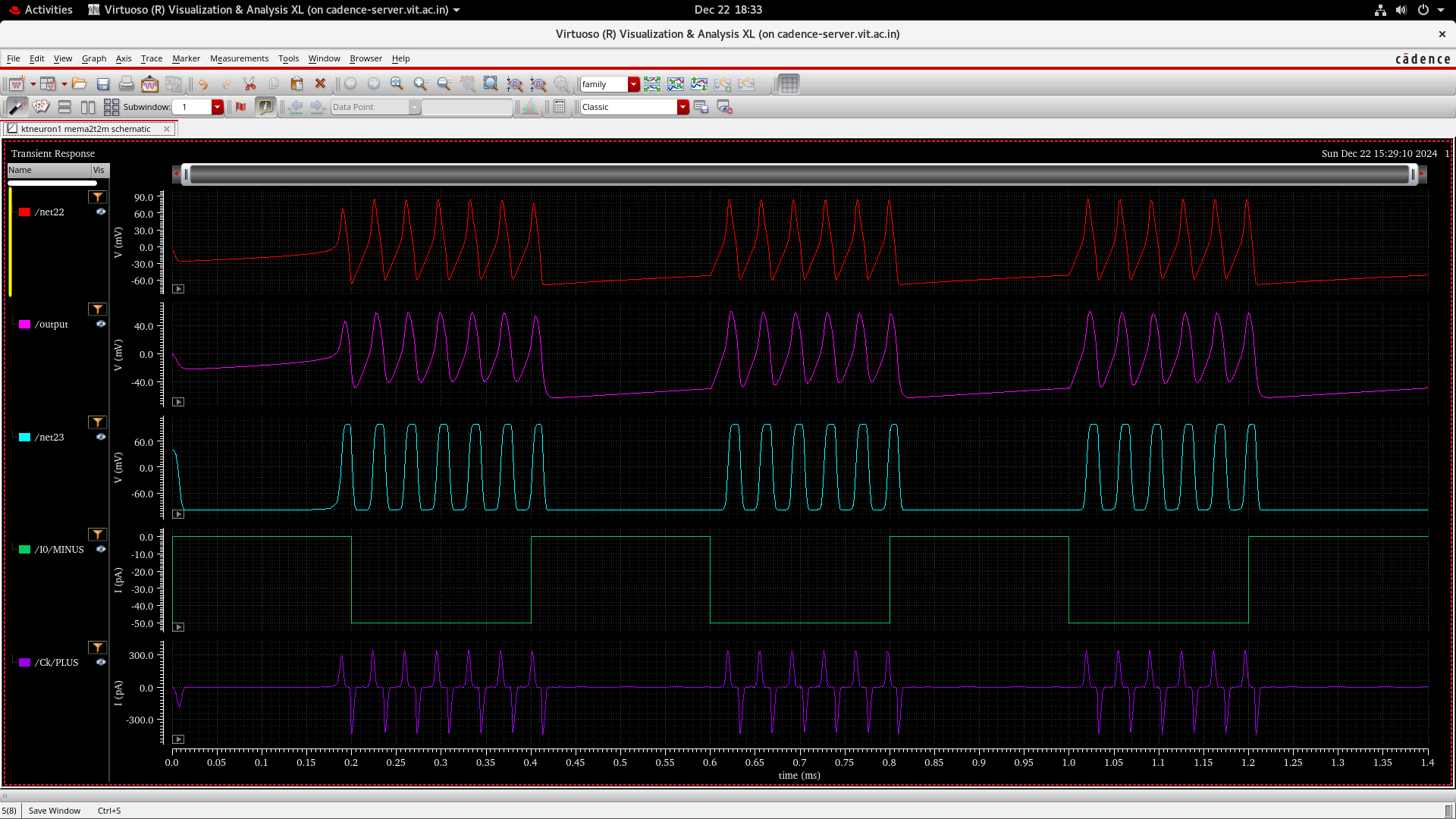

Supplement: S1 Data — (ZIP) [file pone.0318009.s001.zip › Data_avaliabilty/3. Screenshots of cirucits_and_results/Output_waveform_ML neuron with DTDM.png]
